# Control of the Rhizobia Nitrogen-Fixing Symbiosis by Common Bean MADS-Domain/AGL Transcription Factors

**DOI:** 10.3389/fpls.2021.679463

**Published:** 2021-06-07

**Authors:** Litzy Ayra, María del Rocio Reyero-Saavedra, Mariel C. Isidra-Arellano, Luis Lozano, Mario Ramírez, Alfonso Leija, Sara-Isabel Fuentes, Lourdes Girard, Oswaldo Valdés-López, Georgina Hernández

**Affiliations:** ^1^Programa de Genómica Funcional de Eukaryotes, Centro de Ciencias Genómicas, Universidad Nacional Autónoma de México, Cuernavaca, Mexico; ^2^Laboratorio de Genómica Funcional de Leguminosas, Facultad de Estudios Superiores Iztacala, Universidad Nacional Autónoma de México, Tlalnepantla de Baz, Mexico; ^3^Unidad de Análisis Bioinformáticos, Centro de Ciencias Genómicas, Universidad Nacional Autónoma de México, Cuernavaca, Mexico; ^4^Programa de Biología de Sistemas y Biología Sintética, Centro de Ciencias Genómicas, Universidad Nacional Autónoma de México, Cuernavaca, Mexico

**Keywords:** transcription factors, MADS, AGL, common bean, rhizobia, symbiotic nitrogen fixation, nodule

## Abstract

Plants MADS-domain/AGL proteins constitute a large transcription factor (TF) family that controls the development of almost every plant organ. We performed a phylogeny of (*ca*. 500) MADS-domain proteins from Arabidopsis and four legume species. We identified clades with Arabidopsis MADS-domain proteins known to participate in root development that grouped legume MADS-proteins with similar high expression in roots and nodules. In this work, we analyzed the role of AGL transcription factors in the common bean (*Phaseolus vulgaris*) – *Rhizobium etli* N-fixing symbiosis. Sixteen *P. vulgaris AGL* genes (*PvAGL*), out of 93 family members, are expressed – at different levels – in roots and nodules. From there, we selected the *PvAGL* gene denominated *PvFUL*-like for overexpression or silencing in composite plants, with transgenic roots and nodules, that were used for phenotypic analysis upon inoculation with *Rhizobium etli*. Because of sequence identity in the DNA sequence used for RNAi-*FUL-*like construct, roots, and nodules expressing this construct -referred to as RNAi_*AGL*- showed lower expression of other five *PvAGL* genes highly expressed in roots/nodules. Contrasting with *PvFUL*-like overexpressing plants, rhizobia-inoculated plants expressing the RNAi_*AGL* silencing construct presented affection in the generation and growth of transgenic roots from composite plants, both under non-inoculated or rhizobia-inoculated condition. Furthermore, the rhizobia-inoculated plants showed decreased rhizobial infection concomitant with the lower expression level of early symbiotic genes and increased number of small, ineffective nodules that indicate an alteration in the autoregulation of the nodulation symbiotic process. We propose that the positive effects of PvAGL TF in the rhizobia symbiotic processes result from its potential interplay with NIN, the master symbiotic TF regulator, that showed a CArG-box consensus DNA sequence recognized for DNA binding of AGL TF and presented an increased or decreased expression level in roots from non-inoculated plants transformed with OE_*FUL* or RNAi_*AGL* construct, respectively. Our work contributes to defining novel transcriptional regulators for the common bean – rhizobia N-fixing symbiosis, a relevant process for sustainable agriculture.

## Introduction

Transcription factors (TFs) are master control proteins in all living cells, regulating gene expression in response to different stimuli. These exhibit one or more sequence-specific DNA-binding domains that bind to the promoter and/or enhancer regions of multiple target genes to activate or repress transcription. In this way, TF regulates essential biological processes such as development, growth, metabolism, cell cycle progression, and responses to the environment (Czechowski et al., 2004; Libault et al., 2009). An average of 5.7% plant genes code for TF has been distributed among 62 gene families (Libault et al., 2009).

The MADS-domain proteins constitute a eukaryotic family of TFs involved in various relevant functions. Its name derived from the initials of the four founding members of this family *M**INICHROMOSOME MAINTENANCE 1* (*MCM1*) from *Saccharomyces cerevisiae*, which regulates mating type-specific genes, *A**GAMOUS* (*AG*) from *Arabidopsis thaliana* (hereafter referred to as Arabidopsis) and *D**EFICIEN*S (*DEF*) form *Antirrhinum majus*, which act as homeotic factors that control flower development, and *S**ERUM RESPONSE FACTOR* (*SRF*) from *Homo sapiens* which controls serum inducible and muscle-specific gene expression (Schwarz-Sommer et al., 1990; Yanofsky et al., 1990; Pollock and Treisman, 1991; Mead et al., 2002). MADS TF recognizes a consensus DNA sequence called the CArG-box [CC(A/T)_6_GG] (Riechmann et al., 1996).

In plants, the MADS-domain TFs family has expanded largely with 109 members in Arabidopsis, compared with just a few in mammals, *Drosophila melanogaster, Saccharomyces cerevisiae*, and *Caenorhabditis elegans* (Olson et al., 1995; Parenicova et al., 2003). This family of TFs can be divided into two main lineages, type I and type II, based on their protein structure. The best-studied MADS TFs from Arabidopsis and other plant species belong to the type II lineage. Plants type II MADS proteins have a modular domain structure, referred to as the MIKC structure, that contains the N-terminal located (~ 60 amino acids) conserved DNA-binding domain (M), followed by moderately conserved intervening (I) and keratin-like (K) domains, and a variable C-terminal domain (C) that, together with K, have roles for protein complex formation (Alvarez-Buylla et al., 2000b; Smaczniak et al., 2012). These MADS proteins include the regulators of floral transition and floral organ identity determination that, after over 20 years of intensive study, led to the establishment of a general model for flower organ identity in higher plants, the so-called ABCDE model in which floral whorl-specific combinations of class A, B, C, D, or E genes specify floral organ identity (Alvarez-Buylla et al., 2000b; Murai, 2013).

Although initially, *MADS*-box genes were relevant for floral organ speciation, more recent studies have revealed that these genes are relevant for the morphogenesis of almost all plant organs. The role of MADS TF in root development has received relatively less attention, but recently it was comprehensively reviewed by Alvarez-Buylla et al. (2019). About 41 Arabidopsis *MADS*-box genes that show high and intermediate expression levels in different root tissues and zones have been identified (Alvarez-Buylla et al., 2019). Arabidopsis research using genetic, molecular, biochemical approaches has contributed to understanding the role of root-expressed *MADS*-box genes, from different clades, in primary or lateral root development. The latter include *XAL1* (*XAANTAL1*), *XAL2* (*XAANTAL2*), *ANR1* (*Arabidopsis NITRATE REGULATED1*), and *AGL21* (*AGAMOUS-Like21*) (Gan et al., 2005; Garay-Arroyo et al., 2013; Garcia-Cruz et al., 2016). In addition, orthologs of Arabidopsis root *MADS*-box genes from different plant species show similar expression patterns and potential functional conservation. These include *MADS*-box genes from rice (*Oryza sativa*), sweet potato (*Ipomoea batatas*), and *Chrysanthemum morifolium* (Ku et al., 2008; Puig et al., 2013; Sun et al., 2018; Alvarez-Buylla et al., 2019). Studies regarding the expression/function of *MADS*-box genes in legume species are yet scant. *XAL-1* and *XAL-2* genes have been identified from soybean (*Glycine max*) and alfalfa (*Medicago sativa*). These show a high level of expression in the root in soybean, but expression data from alfalfa genes are not available (Alvarez-Buylla et al., 2019). Also, high expression in roots and root hairs has been documented for *AGL21* soybean ortholog (Alvarez-Buylla et al., 2019). Previous work from our group identified orthologs of Arabidopsis root *MADS*-box genes from common bean (*Phaseolus vulgaris*) and soybean that are expressed in root tissues (Íñiguez et al., 2015).

Legumes can establish two types of endosymbiotic associations, with arbuscular mycorrhiza fungi and nitrogen-fixing bacteria, collectively called rhizobia (Olah et al., 2005; Oldroyd, 2013). These associations facilitate the plant acquisition of nutrients such as phosphorus and nitrogen (Oldroyd, 2013; Venkateshwaran et al., 2013).

An efficient symbiotic association with rhizobia results in the formation of root nodules where rhizobia are allocated and fix atmospheric nitrogen (N_2_) informs that the legume host can assimilate in exchange for a carbon source. Symbiotic nitrogen fixation (SNF) is ecologically and economically important for sustainable agriculture because it reduces synthetic fertilizers and the cost of legume cultivation (Venkateshwaran et al., 2013; Castro-Guerrero et al., 2016). The infection of rhizobia to the root tissue is a complex process that involves communication between rhizobia and legumes through molecular signals, such as flavonoids released by the plant root to the rhizosphere that is sensed by compatible rhizobia that, in turn, produce the lipochitooligosaccharides, known as nodulation factors (NF). NF perception by the plant triggers a series of molecular responses essential for rhizobial infection and nodule development (Roy et al., 2020). These responses include the rhizobia-induced root hair deformations or curling required to entrap the rhizobia into an infection chamber and initiate the formation of an infection thread -an invasive invagination of the plant cells- and the infection of the cortical root cells (Suzaki et al., 2015). After rhizobia infection, legumes activate intricate signaling pathways that include finely regulated nodulin genes to develop mature nodules with functionally differentiated bacteroids. Research from the last 20 years has identified a suite of *ca*. 200 legume genes relevant for establishing an effective symbiosis with rhizobia (Roy et al., 2020). The NODULE INCEPTION (NIN) TF gene is a master regulator for the rhizobia symbiosis. It plays an indispensable role in the rhizobial infection and nodule organogenesis symbiotic processes (Schauser et al., 1999; Marsh et al., 2007; Liu and Bisseling, 2020). Recently, it was shown that NIN also plays a major role in the transition from infection into functional nodules, the development of symbiosomes, and nodule senescence (Liu et al., 2021).

Because of the high carbon demand, SNF is an energy-demanding process for the legume host; therefore, the number of infections and the number of nodules per plant is tightly regulated by a systemic feedback regulatory mechanism termed autoregulation of nodulation (AON, Reid et al., 2011; Ferguson et al., 2019). Upon infection, rhizobia induce the root production of CLAVATA3/endosperm surrounding region-related (CLE) peptides, encoded by the *RIC1* and *RIC2* genes in soybean and common bean (Reid et al., 2011; Ferguson et al., 2014). These CLE peptides are transported from the root to the shoot, where they are detected by the nodule autoregulation receptor kinase (NARK) to activate the generation of a shoot-derived signal (i.e., miR2111 and cytokinin) (Tsikou and Yan, 2018; Ferguson et al., 2019). The shoot signals are transported to the root to inhibit further nodule development through the participation of the negative regulator TML (Too Much Love), a kelch repeat-containing F-box protein (Takahara et al., 2013). By interplay with other regulators, the NIN TF plays a relevant role in the AON process through the transcriptional activation of the CLE peptides (Soyano et al., 2014; Roy et al., 2020).

The information about legume *MADS*-box genes as regulators of the legume -rhizobia symbiosis is scarce. Three *MADS*-box genes from alfalfa (*Medicago sativa*) from the *AGL17* subfamily with high expression in nodules were cloned (*nmh7, nmhC5, ngl9*) (Heard et al., 1997; Zucchero et al., 2001). Expression analysis of the alfalfa *NMH7* gene revealed its expression in flowers and nodules and the parenchyma or the root tip and the root elongation zone from seedlings (Páez-Valencia et al., 2008). The soybean *GmNMHC5* gene is highly expressed in roots and nodules, and its overexpression promotes lateral root development and nodulation (Liu et al., 2015). Contrastingly, the *GmNMH7, MADS-box* gene, highly expressed in roots and nodules, exerts negative control to nodulation, probably through regulating the content of gibberellin (Ma et al., 2019).

Common bean (*Phaseolus vulgaris*) is the most important crop legume for human consumption and the principal source of non-animal protein for human consumption in the developing world (Broughton et al., 2003). Research from our group focused on the common bean – rhizobia symbiosis has contributed to identifying microRNAs/TF target gene nodes that play a relevant role in the regulation of the SNF, such as miR172c/AP2-1 and miR319d/TCP10 (Nova-Franco et al., 2015; Martin-Rodriguez et al., 2018). We have also identified common bean and soybean *MADS*-box genes expressed in roots, similar to their Arabidopsis orthologs (Íñiguez et al., 2015). This work aimed to analyze the role of *MADS*-box genes from *P. vulgaris*, hereafter denominated *PvAGL* genes, in the common bean – rhizobia symbiosis that, to our knowledge, have not been studied in this legume. We performed a phylogenetic analysis of *MADS*-box genes for Arabidopsis and four legume species that identified clades, including root-expressed MADS-box genes from Arabidopsis and legumes. Expression analysis revealed that *PvAGL* genes are highly expressed in both rhizobia-inoculated roots and nodules. The functional analysis of composite *Rhizobium etli*-inoculated common bean plants with a modulated expression of *PvAGL* genes revealed the participation of TF from this gene family in rhizobial infection, expression of early symbiotic genes, root architecture, nodulation, nitrogenase activity, and the AON symbiotic process. Our work contributes to the knowledge of the participation of members from the *MADS*-box/*AGL* TF family in the legume – rhizobia SNF, a relevant process for sustainable agriculture.

## Materials and Methods

### Phylogenetic Analysis

Gene sequences and the RNA-seq gene expression data for the *MADS*-box TFs genes for each plant species were retrieved from the following databases. For Arabidopsis, The Arabidopsis Information Resource TAIR (https://www.arabidopsis.org); for *P. vulgaris* the Gene Expression Atlas (GEA), *Pv* GEA (http://plantgrn.noble.org/PvGEA/) (O'Rourke et al., 2014); for *G. max* the ePlant BAR page (http://bar.utoronto.ca/eplant_soybean/) (Waese et al., 2017), for *Medicago truncatula* the MtGEA (https://mtgea.noble.org/v3/) and *Lotus japonicus* the Lotus Base (https://lotus.au.dk/).

The translated protein sequences from the *MADS-*box genes were analyzed in Interpro (http://www.ebi.ac.uk/interpro/) to confirm the presence of the characteristic domain IPR002100 from this gene family. Next, the protein sequences were analyzed with blast v2.6.0 and aligned with clustalw v2.1 (Thompson et al., 1994) to confirm that proteins share a stereotypical MIKC structure with four domains characteristic of type II *MADS*-box genes of the plant (Alvarez-Buylla et al., 2000b; Smaczniak et al., 2012). Finally, the proteins with MIKC structure were re-analyzed by multiple alignments to verify the integrity of the MADS domain (~60 amino acidic region). Some protein sequences from each species were excluded for phylogenetic analysis because these did not have a complete MADS domain sequence. The whole set of 511 MADS protein sequences from Arabidopsis and four legume species used for phylogenetic analysis are listed in the Supplementary Table 1.

The best evolutionary model (JTT+G) was determined with Prottest3 v3.4.2, and a maximum likelihood phylogeny was made with PhyML v3.0 using approximate likelihood-ratio test SH-like as a statistical test for branch support. Finally, the Interactive Tree of Life (iTOL5) (Letunic and Bork, 2019) was used for displaying the phylogenetic tree.

### Plant Material and Growth Conditions

The common bean (*P. vulgaris*) Mesoamerican cv BAT93 was used in this work (Vlasova et al., 2016). Seeds were surface sterilized in 10% (V/V) commercial sodium hypochlorite for 10 min and finally rinsed three to four times in sterile distilled water. Then seeds were germinated on sterile moistened filter paper at 30°C in darkness for 2 days. Germinated seedlings were planted in pots with wet sterile vermiculite. For SNF conditions, plantlets were inoculated with 1 ml saturated liquid culture of the *Rhizobium etli* CE3 strain per plant. Plants were grown in growth chambers under controlled environmental conditions (25–28°C, 16 h photoperiod) and were watered every 3 days with N-free B&D nutrient solution (Broughton and Dilworth, 1971). For non-inoculated conditions, a full nutrient B&D solution (5 mM N-content) was used to water the plants. Common bean composite plants with transgenic roots were generated as described below and grown in sterile pots with perlite to prevent harming the transgenic root/nodules when taking these out of the pot at harvest time. The growth conditions of composite plants were similar to those described for wild-type plants.

### RNA Isolation and Quantitative RT-PCR Analysis

Total RNA was isolated from frozen tissues collected directly into liquid nitrogen and stored at −80°C. The wet weight used for RNA isolation from each tissue was: 100 mg for nodules detached from roots, 250 mg for roots, and 200 mg for leaves. Trizol^TM^ Reagent (Thermo Fischer Scientific, Inc., Waltham, MA, USA) was used, following the manufacturer's instructions. Total RNA was quantified using the NanoDrop spectrophotometer (Thermo Fischer Scientific, Inc.). For quantification of transcripts levels, total RNA (2 μg) was treated with DNaseI RNase-free (Thermo Fischer Scientific, Inc., Waltham, MA, USA) to remove genomic DNA. First, strand cDNA was synthesized using RevertAid H Minus First Strand cDNA Synthesis Kit (Thermo Fisher Scientific, Inc., Waltham, MA, USA). The resulting cDNAs were then diluted and used to perform qRT-PCR assays using SYBR Green PCR Master Mix (Thermo Fischer Scientific, Inc.), following the manufacturer's instructions. The sequences of oligonucleotide primers used for qRT-PCR of each gene are provided (Supplementary Table 2). Assays were run in 96-well plates using the 7300 Real-Time PCR System and 7300 System Software (Applied Biosystems, Foster City, CA, USA) with settings of 50°C for 2 min, 95°C for 10 min, and 40 cycles of 95°C for 15 s and 57°C for 60 s. Relative expression for each sample was calculated using the “comparative Ct method” and normalized with the geometrical mean of three housekeeping genes (HSP, MDH, and UBQ9) (Vandesompele et al., 2002). Student's *t*-test was performed to evaluate the significance of the differential expression using the mean values from three biological replicates for each condition, using the GraphPad Prism v8.0 for Windows (GraphPad Software, San Diego, CA, USA).

### Plasmid Construction, Plant Transformation, and Generation of Composite Plants

For overexpression of the Phvul.008G027800.1 *P. vulgaris MADS*-box gene, hereafter denominated *PvFUL-*lik*e* (Phvul.008G027800) based on its ortholog Arabidospis *FUL* (*FRUITFULL*), was PCR-amplified using as template cDNA from common bean roots and the specific primers Up_*FUL* 5′-CCCTCGAGCTTTTCCACAATTGCC-3′ and Lw_*FUL* 5′-GCCCGGATCCTAACTAGTAAGTAG-3′. The purified PCR product (767 bp) was cloned into the pTOPO2.1 intermediate vector and confirmed by sequencing. To construct the OE_*FUL* plasmid, the *PvFUL*-like DNA region was excised using XhoI/BamHI sites and cloned into the pTDTO plasmid (Aparicio-Fabre et al., 2013). This expression plasmid contains the 35S cauliflower mosaic virus (35SCaMV) promoter and the tdTomato (red fluorescent protein) gene as a visible reporter gene. For silencing *PvAGL* genes, by RNAi strategy, specific primers flanking a DNA region coding for the MADS-domain from the *PvFUL*-like gene (Up_RNAi 5′-TCAGCTCAAGCGGATCG-3′ and Lw_RNAi 5′-CACCACGTTCCAAGACATCTT-3′) were used to amplify a 194 bp fragment using as template cDNA from common bean roots. This DNA fragment, that share homology among *PvAGL* genes highly expressed in roots/nodules, was cloned by the Gateway system into the intermediate vector pENTR and finally in the pTDT-DC-RNAi plasmid (Valdes-Lopez et al., 2008), which also contains the 35S cauliflower mosaic virus (35SCaMV) promoter and the tdT (tandem double Tomato, red fluorescent protein) gene as a visible reporter gene.

The empty vectors pTDTO and pTDT-DC-RNAi, hereafter denominated EV, and the resulting plasmids OE_*FUL* and RNAi_*AGL* were introduced by electroporation into *Agrobacterium rhizogenes* K599, which was then used for plant transformation as described (Estrada-Navarrete et al., 2007) with minor modifications (Aparicio-Fabre et al., 2013). In addition, the presence of red fluorescence from the tdTomato reporter gene was routinely checked in the putative transgenic roots/nodules using a fluorescence stereomicroscope.

### Identification of Putative *Cis*-Regulatory Elements in *P. vulgaris* Genes

DNA sequences from the region upstream of the initiation codon were retrieved from Phytozome v12.1 (https://phytozome.jgi.doe.gov/pz/portal.html). The length of the intergenic 5′-upstream sequences analyzed was 20 kbp or shorter depending on the position of the next contiguous gene. For *PvFUL-*like gene 7.3-kb sequence was analyzed using the New PLACE tool (A Database of Plant *Cis*-acting Regulatory Elements; https://www.dna.affrc.go.jp/PLACE/?action=newplace) (Higo et al., 1999) to identify putative *cis*-regulatory elements related to nodulation or root development; these are shown in Supplementary Figure 2. Upstream sequences of *P. vulgaris* early symbiotic genes were analyzed using SnapGene Viewer (GSL Biotech LLC, Chicago, IL, USA; available at snapgene.com) to identify the consensus CArG-box sequence that is recognized by AGL TF (Riechmann et al., 1996).

### Root Hair Deformation Analysis

Common bean composite plants, expressing the empty vectors (EV) or OE_*FUL*/RNAi_*AGL* plasmids and growing in 25 cm × 25 cm Petri dishes containing nitrogen-free Fahräeus medium (Catoira et al., 2000), were inoculated with 1 mL of saturated *Rhizobium etli* CE3 culture (OD_600_ = 1). Five days after inoculation, tdTomato-positive transgenic roots were collected. The root-susceptible zone for rhizobial infection covered around 2 cm was stained with methylene blue for 1 h to maximize contrast and washed three times with double-distilled water. The quantification of the number of rhizobia-induced root hair deformations was determined from 1 cm root segments from the susceptible zone. Deformation events were observed with a bright-field microscope equipped with an 18 MP Digital Camera with Aptina CMOS Sensor (Cientifica Vela Quin, Iztapalapa, Mexico city, Mexico). A total of 20 independent biological replicates were generated, each one including 10 plants.

### Phenotypic Analysis and Nodule Histology

For root length, root area, and nodule perimeter evaluations, pictures from root or nodule tissues of composite plants (18 or 28 dpi) were processed with SmartRoot software (Lobet et al., 2011).

Determination of the Nase-enzyme activity was determined in nodulated roots (28 dpi) by acetylene reduction assay (ARA) described by Hardy et al. (1968). Specific activity is expressed as nmol ethylene h^−1^/plant.

For histological analysis, nodules (28 dpi) were collected from composite plants transformed with EV or OE_*FUL*/RNAi_*AGL* plasmids and were treated with the procedure described by Reyero-Saavedra et al. (2017). In addition, representative images from safranine-stained sections (25 μm) were taken with a NIKON camera coupled to a bright-field microscope.

### Statistical Analyses

The graphs and statistical analysis were made with the R software 3.0.1 or the GraphPad Prism v8.0. The specific statistical tests performed are indicated in the legend of the corresponding figures.

## Results

### Phylogeny of the MADS-Domain TF From Arabidopsis and Legume Plants

**Figure 1:**
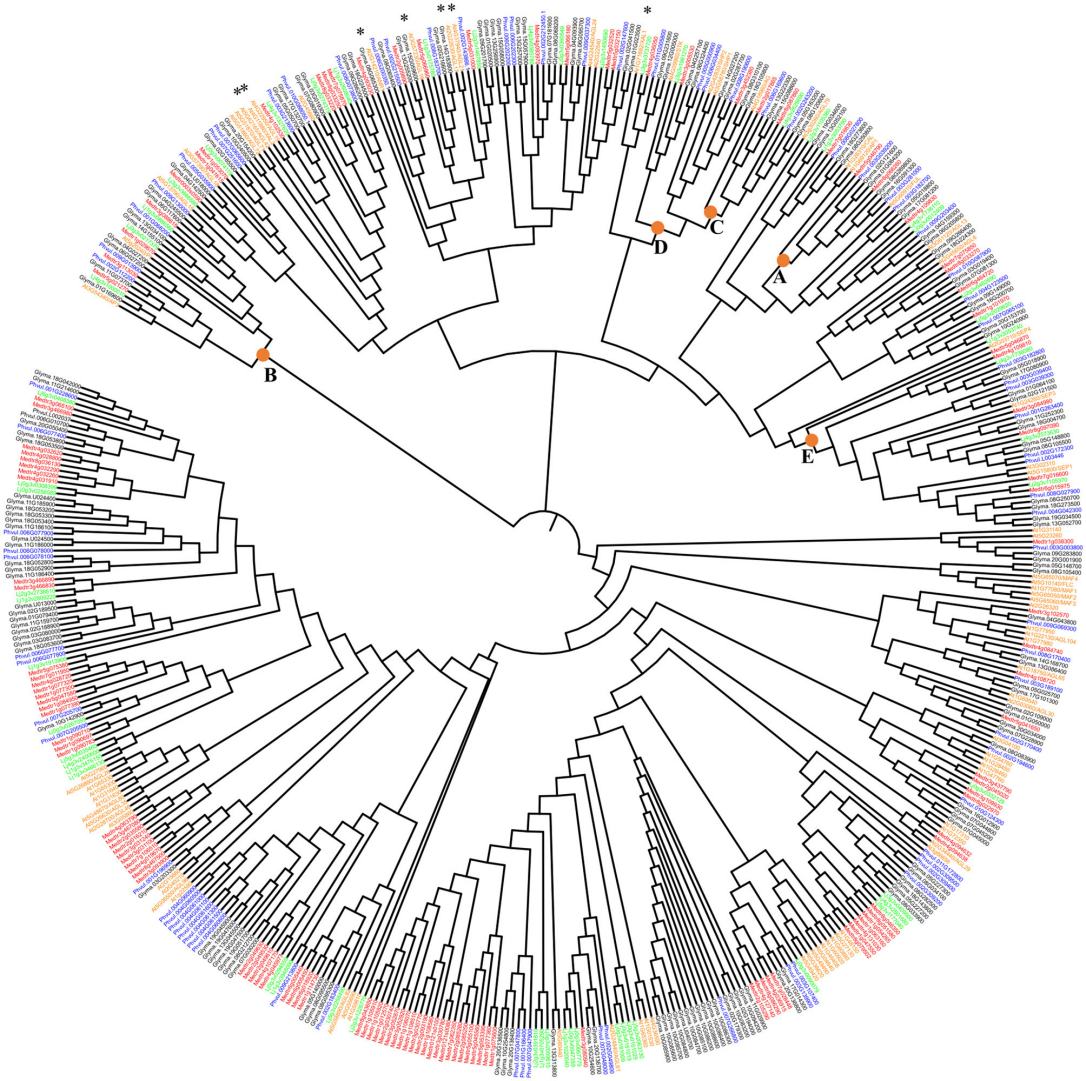
Maximum likelihood phylogenetic tree of the AGL-TFs from *Phaseolus vulgaris* (blue), *Glycine max* (black), *Medicago truncatula* (red), *Lotus japonicus* (green), and *Arabidopsis thaliana* (orange). Stars highlight those Arabidopsis *AGL* genes highly expressed in roots that are analyzed in Figure 2 together with legumes' genes from those clades. SH-like branch support values (not shown) were higher than 0.70 in 80% of the phylogeny branches. The nodes including clades A, B, C, D, or E class MADS-domain proteins from Arabidopsis are indicated Support values for these clades were 0.87, 0.84, 0.85, 0.85, and 0.84, respectively.

In plants, the *MADS-*box is a large gene family of TF. The number of family members varies in different plant species. In this work, we identified *MADS-*box genes for four legume species. For *P. vulgaris* (common bean), we identified 93 *AGL* genes, for *G. max* (soybean) 183, for *M. truncatula* (Medicago) 143, and *L. japonicus* (Lotus) 79. For the phylogenetic analysis of MADS TF from Arabidopsis and four legume species, those MADS protein sequences that did not show a complete MADS domain sequence were excluded. Thus, a 511 MADS-domain protein sequences set (Supplementary Table 1) was used for the maximum likelihood phylogenetic tree shown in Figure 1.

**Figure 2:**
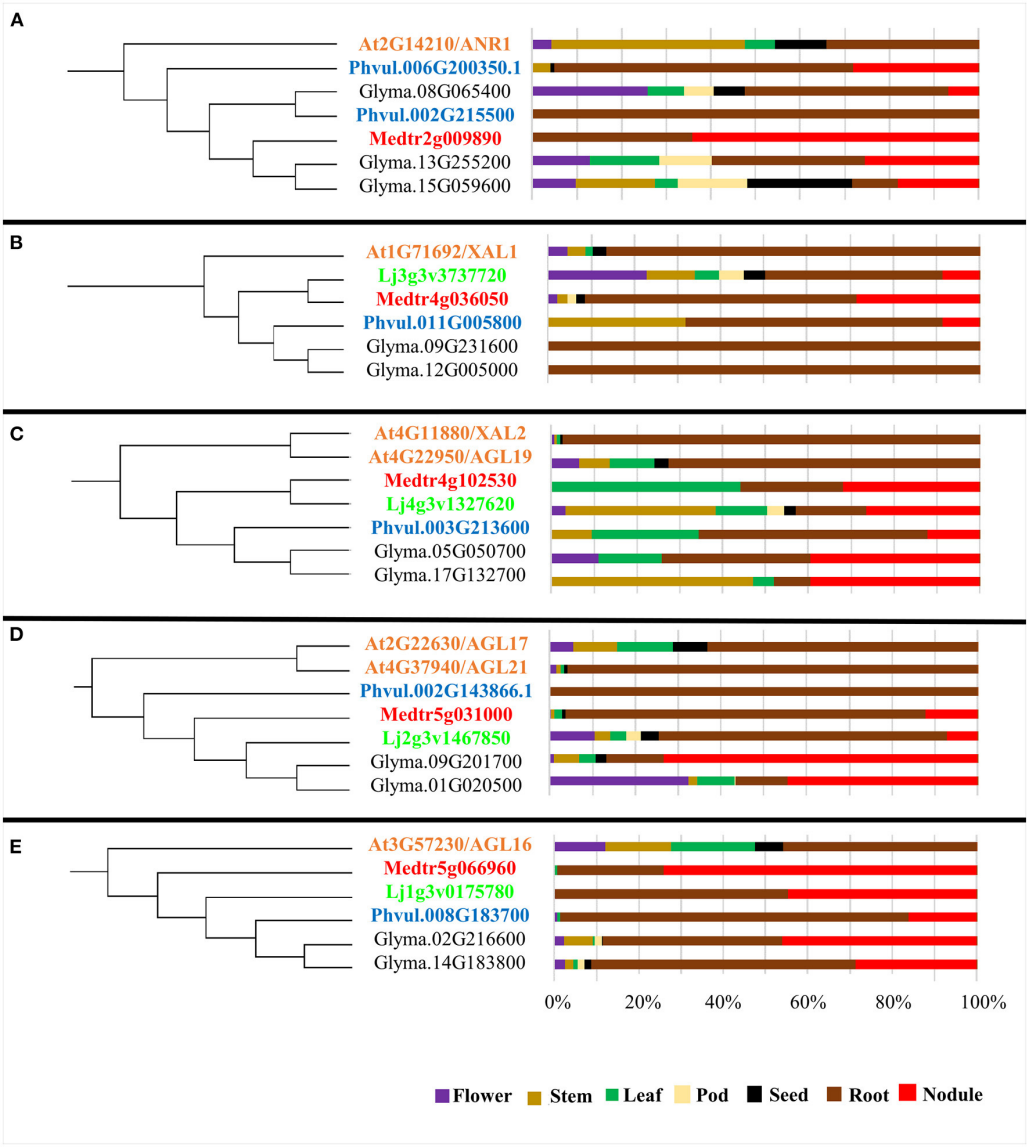
Expression profile by tissue of *AGL* genes from different plant species grouped in clades including Arabidopsis *AGL*s highly expressed in roots. **(A–E)** The left side of each panel shows one selected clade (highlighted in Figure 1); the color code used for genes from each species is the same as in Figure 1. The right side of each panel shows the % expression level in each tissue (flower, purple; stem, ocher; leaf, green; pod, beige; seed, black; root, brown and nodule, red) of the corresponding *AGL* gene. As indicated in the Material and methods section, expression level values were extracted from the Gene Expression Atlas for each species.

The majority of clades from the phylogenetic tree (Figure 1) group MADS from Arabidopsis and the four legumes species analyzed, suggesting that MADS TF from the same clade share their tissue expression and their participation in regulatory networks from a certain vegetative tissue. In Figure 1, we indicated seven Arabidopsis root-expressed *MADS*-box genes from five different clades involved in regulating root development (Alvarez-Buylla et al., 2019). We assessed if the legume *MADS*-box genes grouped in these monophyletic clades were also expressed in root and if, additionally, these were expressed in root-nodules elicited by rhizobia. The clade from Figure 2A includes *ANR1 (ARABIDOPSIS NITRATE REGULATED 1*; (Gan et al., 2005) and six legume *MADS*-box genes with high expression in root tissues, and five of these also showed expression in nodules. The Arabidopsis *XAL1* (Garcia-Cruz et al., 2016) clade (Figure 2B) groups five legume genes that showed enhanced expression in roots, and three of these also showed expression in nodules. The clade depicted in Figure 2C includes *XAL2* and *AGL19* (Alvarez-Buylla et al., 2000a, 2019; Garay-Arroyo et al., 2013) as well as five legume *MADS*-box genes that were expressed in root and nodules. Figure 2D shows the clade with *AGL17/21* genes (Burgeff et al., 2002; Alvarez-Buylla et al., 2019) grouped with five legume genes that showed expression in roots, and four of these were also expressed in nodules. The clade from Figure 2E includes the *AGL16* (Nawy et al., 2005) and five legume genes whose expression in roots or nodules was >50%, compared with the expression level from other organs.

### Expression Analysis of *PvAGL* Genes in Root/Nodule

Previous work from our group identified the *MADS*-box genes from common bean and soybean, an ortholog of root-expressed Arabidopsis *MADS*-box genes that are expressed in roots and nodules (Íñiguez et al., 2015). Our previous report (Íñiguez et al., 2015) and data presented in Figures 1, 2 prompted us to propose that *P. vulgaris AGL* genes highly expressed in roots/nodules participate as regulators of the common bean - rhizobia symbiosis. In this work, we updated the analysis of *PvAGL* genes based on the recent annotation of the *P. vulgaris* genome sequence (www.phytozome.net/commonbean.php) and the identification of the total number of *PvAGL* genes from this work (Figure 1). RNA-seq data analysis revealed 16 *PvAGL* genes, out of 93 gene family members, with expression in root and nodule tissues compared with their expression in the stem, leaf, pod, and seed tissues (Supplementary Figure 1). To assess the reliability of the RNA-seq data, we performed qRT-PCR gene expression analysis from root, nodules, and leaves tissues and confirmed that these *PvAGL* genes are expressed in root/nodules, albeit at different levels (Supplementary Figure 1).

**Figure 3:**
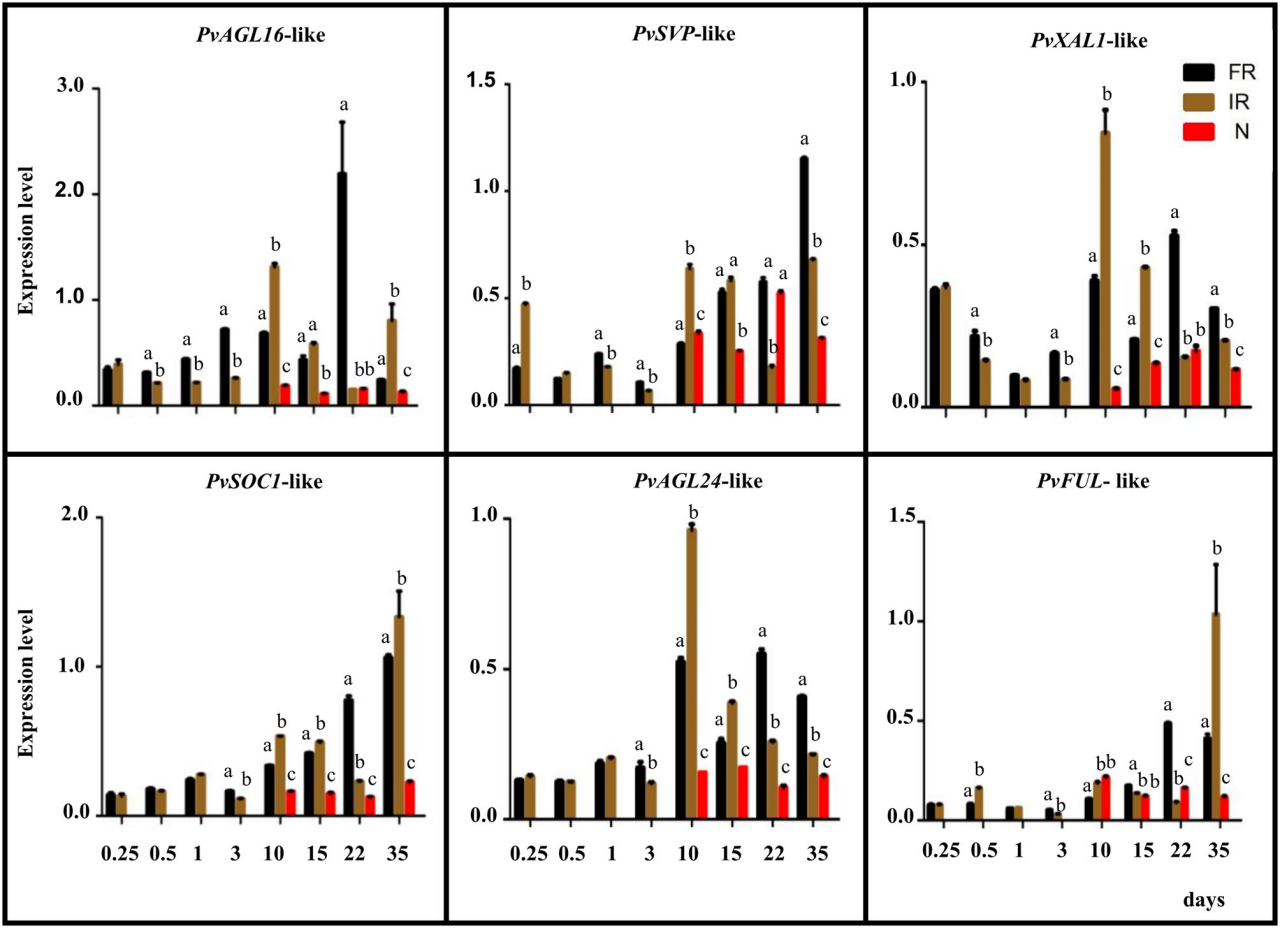
Expression analysis of *P. vulgaris AGL* genes highly expressed in root/nodules. Expression levels were determined by qRT-PCR in roots or nodules harvested at the indicated time points, corresponding to days post-inoculation for *R. etli*-inoculated plants or days after planting for non-inoculated plants. NR: non-inoculated roots, black histograms. IR: inoculated roots, brown histograms. N. nodules, red histograms. Expression level refers to gene expression, based on Ct value, normalized with the expression of the housekeeping genes. The name assigned to each *P. vulgaris AGL* gene corresponded to its Arabidopsis ortholog. The gene IDs are shown in Supplementary Figure 1. Values from each time point for each gene were statistically analyzed by Student's *t*-test, in the case of two samples, or one-way analysis of variance (ANOVA), in the case of three samples (*p*-value < 0.001). Different lower-case letters indicate statistically different groups.

We pursued the expression analysis of six *PvAGL* genes that showed the highest expression in root/nodule tissues (Supplementary Figure 1) by assaying their transcript levels from these tissues of *R. elti*-inoculated plants as well as from root of non-inoculated plants at different developmental stages. According to our phylogenetic analysis, the name we assigned to each PvAGL gene corresponds to its Arabidopsis ortholog (Figure 1). Figure 3 shows expression level values for *PvAGL16*-like, *PvSVP*-like (*SHORT VEGETATIVE PHASE*), *PvXAL1-*like*, PvSOC1-*like *(SUPPRESSOR OF OVEREXPRESSION OF CONSTANS 1), PvAGL24-*like, and *PvFUL*-like (*FRUITFULL*). The six genes analyzed showed significantly lower expression in young roots compared with increased levels in roots from later developmental stages, except for *PvXAL1*-like that showed high expression in 12-h roots (Figure 3). These genes were also expressed in immature (10–15 dpi), mature (22 dpi), and senescent (35 dpi) nodules, albeit at a lower level than in non-inoculated or inoculated roots, except for *PvFUL-*like (Figure 3).

Interestingly, the transcript level of the *PvFUL*-like gene in rhizobia-inoculated plants was similar in roots compared with immature nodules and was higher in mature nodules compared with roots. In contrast, at nodule senescence, the expression in roots was highly increased (Figure 3). Furthermore, the *cis*-elements *in silico* analysis of *PvFUL*-like gene promoter sequence (Supplementary Figure 2) revealed the presence of the organ-specific elements OSE1ROOTNODULE (Vieweg et al., 2004) and OSE2ROOTNODULE (Fehlberg et al., 2005) with characteristic consensus sequences motifs of the promoters activated in infected cells of root nodules. These were repeated 9 and 18 times, respectively, among the analyzed sequence (Supplementary Figure 2). In addition, one *cis*-element related to root development, repeated 39 times, was also identified (Supplementary Figure 2).

### Effect of PvAGL TF in Rhizobia-Infection of *R. etli*-inoculated Common Bean Plants

To further investigate the regulatory role of root/nodule-expressed *PvAGL* TF genes in the common bean-rhizobia N-fixing symbiosis, we aimed to modulate their expression in common bean composite plants – with transgenic root/nodules and untransformed aerial organs – generated through *A. rhizogenes*-mediated transformation (Estrada-Navarrete et al., 2007).

The *PvFUL*-like gene was selected for the over-expression construct (OE_*FUL*) and the silencing construct (RNAi_*AGL*) driven by the 35SCaMV promotor. These constructs and the empty control vector (EV) contained the tdTomato (red fluorescent protein) reporter gene (Valdes-Lopez et al., 2008; Aparicio-Fabre et al., 2013). As expected, a very high *PvFUL*-like transcript level was expressed in transgenic roots and nodules from composite plants transformed with OE_*FUL* (Supplementary Figure 3). The (194 bp) cDNA fragment from the *PvFUL*-like gene used for the RNAi construct (Supplementary Figure 4) codes for the *MDS*-box sequence. Multiple sequence alignment analysis of this *PvFUL*-like gene sequence with a corresponding sequence of *PvAGL* genes showed similar sequence identity, ranging from *ca*. 50–70% (Supplementary Figure 4). Gene expression level from the six *PvAGL* genes highly expressed in root/nodules (Figure 3) was determined from transgenic roots and nodules expressing the EV or the RNAi constructs (Supplementary Figure 5A). Compared with control EV tissues, the roots and nodules expressing the silencing construct have a significantly decreased expression level of PvFUL-like gene and *PvXAL1*-like*, PvSVP*-like, *PvSOC1*-like and *PvAGL16*-like, and *PvAGL24*-like. Gene silencing in nodules was high, showing a very low expression level; generally, the silencing effect in roots was lower than in nodules (Supplementary Figure 5A). In agreement with published GEA data (O'Rourke et al., 2014; Íñiguez et al., 2015) and data from Supplementary Figure 1, we confirmed very low, negligible, expression of other 10 *PvAGL* genes in EV and RNAi_*AGL* roots/nodules (Supplementary Figure 5B). The simultaneous silencing of several *PvAGL* genes in transgenic roots/nodules interpreted as a result of the homology among *MADS*-box cDNA sequence cloned for the RNAi silencing strategy would avoid functional redundancy among these genes. It may result in highly altered phenotypes of RNAi_*AGL* plants (see below).

**Figure 4:**
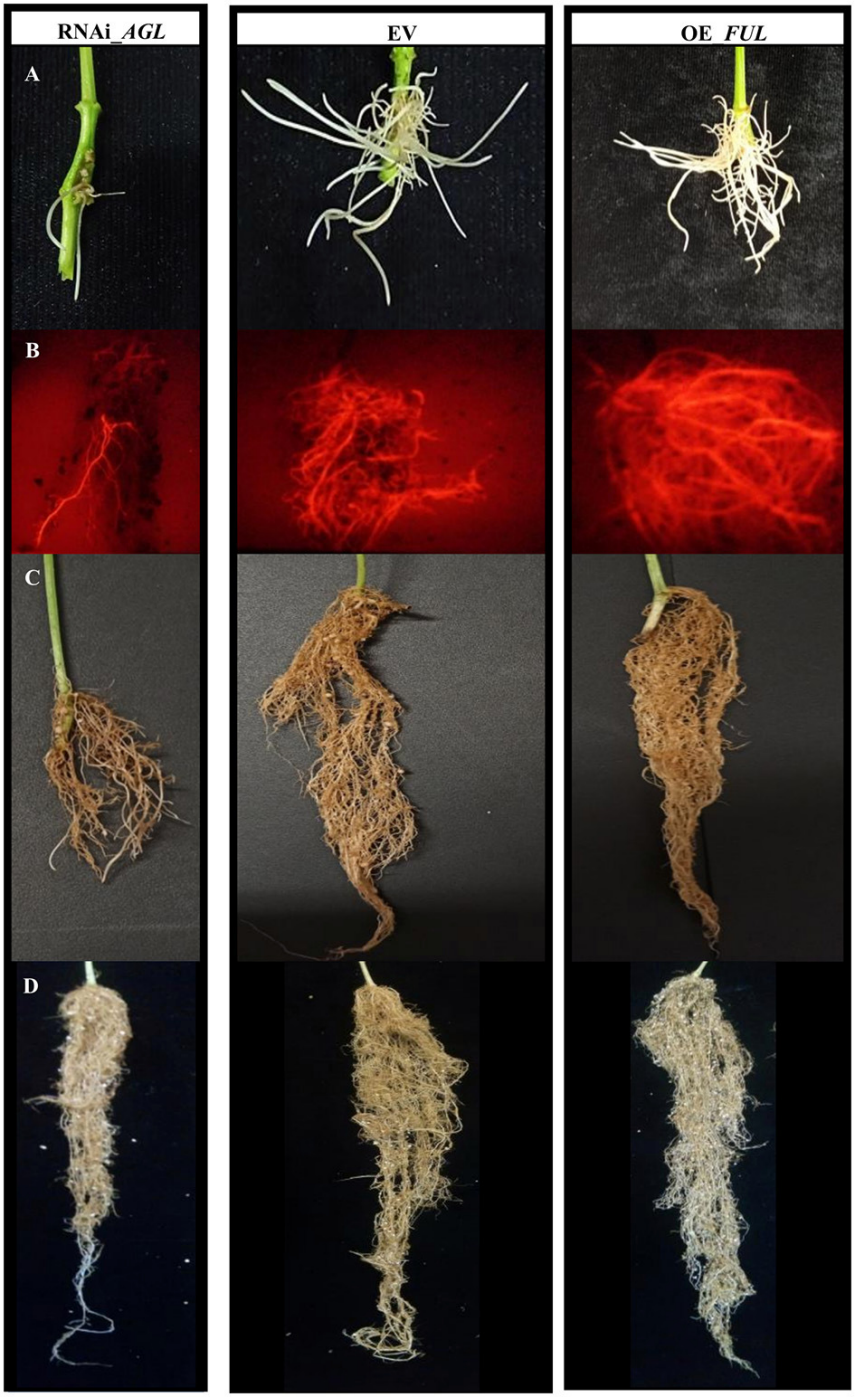
Common bean transgenic roots expressing the RNAi_*AGL*, EV, or OE_*FUL* constructs. **(A)** Emerging transgenic roots, 12 days after *A. rhizogenes* transformation. **(B)** Fluorescent image showing transgenic expression of red fluorescent protein (tdT) in roots from *R. etli*-inoculated composite plants, at 18 dpi. **(C)** Roots from *R. etli*-inoculated composite plants at 28 dpi. **(D)** Roots from non-inoculated composite plants at 7 days after planting. Representative images are shown.

Common bean plantlets transformed with *A. rhizogenes* bearing the RNAi_*AGL* developed fewer and shorter hairy roots, emerging from the infection site, instead of those transformed with OE_*FUL* or EV that showed numerous and longer hairy roots (Figure 4A). In addition, the RNAi-*AGL* composite plants grown under non-inoculated conditions showed reduced root growth (Figure 4D).

**Figure 5:**
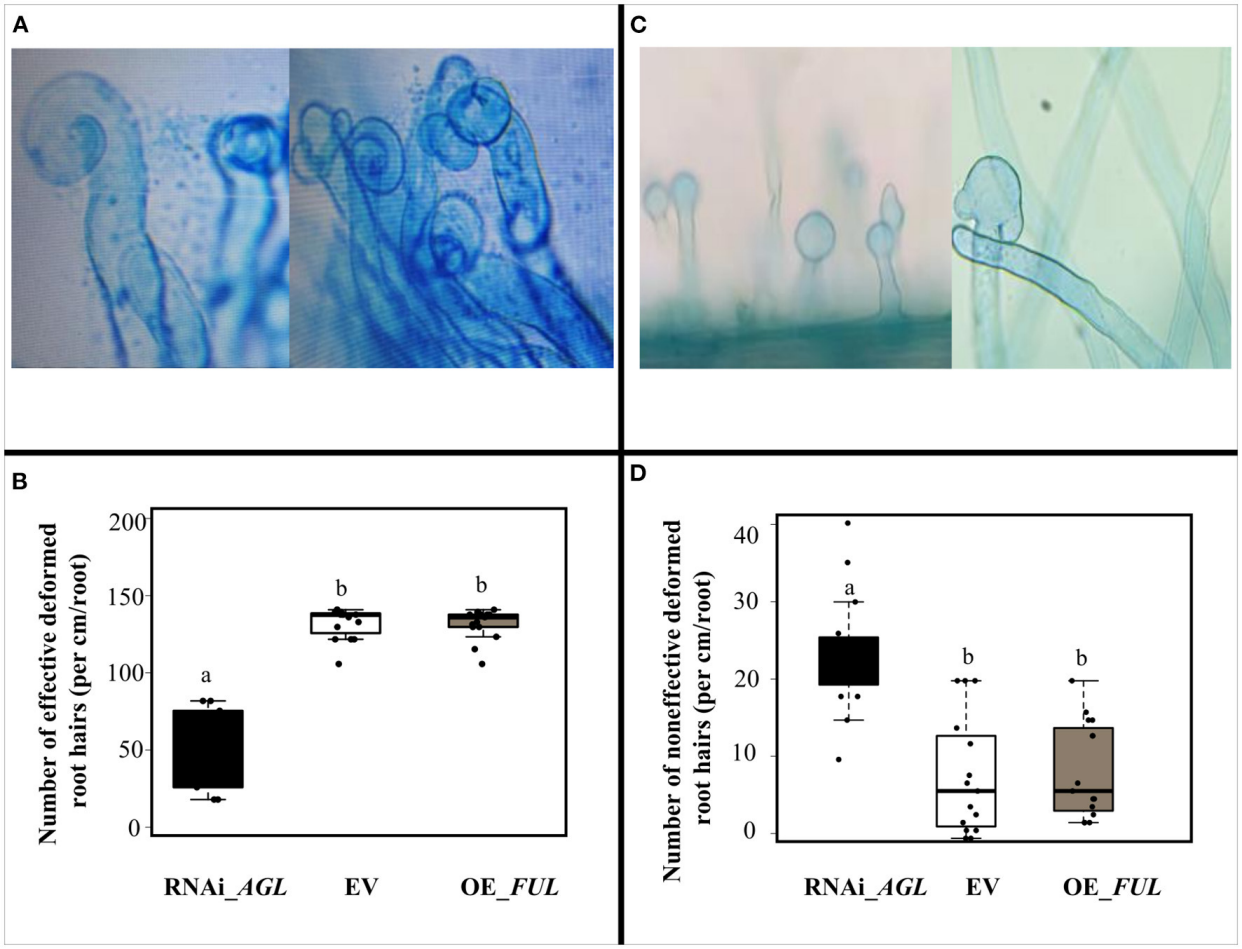
Response to rhizobial infection of transgenic roots expressing the RNAi _*AGL*, or OE_*FUL* construct compared with the control vector (EV). **(A)** Representative images of characteristic rhizobia-induced root hair deformation in common bean transgenic roots. **(B)** The number of effective rhizobia-induced root hair deformation in transgenic roots expressing each construct. **(C)** Representative images of spatula-like deformed root hair observed in RNAi_*AGL* silenced roots. **(D)** The number of aberrant rhizobia-induced root hair deformations in transgenic roots expressing each construct. Data shown were obtained from 5-days old seedlings and inoculated with *R. etli* as described in the Material and Methods section. The number of rhizobia-induced root hair deformation events were quantified per cm/root from the susceptible zone from 20 different common bean roots. The horizontal box side represents the first and third quartile in box plots while the outside whiskers the minimum and maximum values. Different lower-case letters indicate a significant difference according to one-way analysis of variance (ANOVA) (*p*-value < 0.001).

To assess if the negative effect of *PvAGL* silencing in hairy root formation/growth also affects the common bean symbiosis with rhizobia, we first analyzed rhizobial infection to young transgenic roots inoculated with *R. etli* CE3. No significant differences in root hair development nor root hair density were observed in control (EV) plants than roots with modulation of *PvAGL*-gene expression (Supplementary Figure 6). Next, we quantified the root hair deformation upon rhizobia infection (Figure 5). Notably, inoculated roots expressing the RNAi_*AGL* construct showed significantly fewer effective deformed or curled roots hairs (Figures 5A,B) that form an infection chamber, where rhizobia are trapped would allow the formation of the infection thread (Fournier et al., 2015). In addition, *PvAGL*-silenced roots showed an increased number of non-effective or spatula-like (Reyero-Saavedra et al., 2017) root hair deformations (Figures 5C,D). The inoculated roots overexpressing the *PvFUL*-like gene showed a similar number of curled root hair than control EV inoculated roots (Figure 5B).

**Figure 6:**
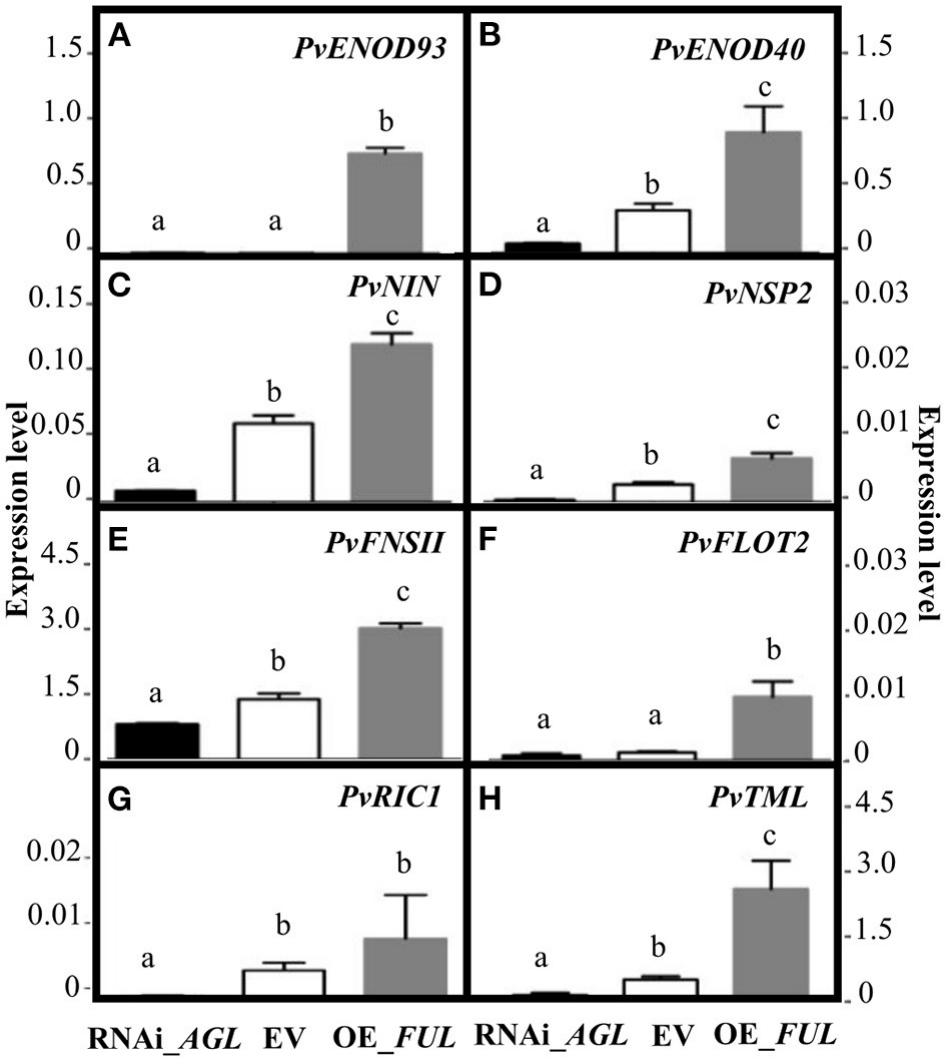
Expression levels of early nodulation genes upon *R. etli* infection of transgenic roots expressing the RNAi _*AGL* or OE_*FUL* construct compared with EV. The common bean early nodulation genes were evaluated at 2 dpi, these were **(A)** *PvENOD93* (Phvul.003G162150), **(B)** *PvENOD40* (Phvul.002G064166), **(C)** *PvNIN* (Phvul.009G115800), **(D)** *PvNSP2* (Phvul.009G115800), **(E)** *PvFNSII* (Phvul.003G074000), **(F)** *PvFLOT2* (Phvul.009G090700), **(G)** *PvRIC1* (Phvul.005G096900), and **(H)** *PvTML* (Phvul.001G09440). Expression levels refer to gene expression, based on Ct value, normalized with the expression of the housekeeping *UBC9* gene. Different lower-case letter columns indicate significant difference, according to one-way analysis of variance (ANOVA) (*p*-value < 0.001). Data showed obtained from three biological replicates, and one technical replicate each from common bean plants.

The fact that the silencing of Pv*AGL* genes negatively affected the rhizobia infection process led us to hypothesize that the Pv*AGL* TFs regulate, either directly or indirectly, the expression of key symbiosis-related genes. To test this hypothesis, we evaluated the expression level of *PvENOD93 (EARLY NODULIN 93), PvENOD40 (EARLY NODULIN 40), PvNIN, PvNSP2 (NODULATION SIGNALING PATHWAY 2), PvFNSII (FLAVONE SYNTHASE II)*, and *PvFLOT2 (FLOTILLIN 2)* genes from RNAi_*AGL*, OE_*FUL* and EV roots inoculated with *R. etli* for 2 days. The data presented in Figure 6 showed that, compared with gene expression level in EV control roots, every gene evaluated showed reduced transcript level in RNAi-*AGL* roots but an increased transcript level in OE_*FUL* roots. Thus, there was a correlation between altered root hair deformation and the expression of early symbiotic genes essential for rhizobial infection (Figures 5, 6). Furthermore, to infer if these *P. vulgaris* early symbiotic genes could be direct targets of PvAGL transcriptional regulation, we searched for the consensus DNA sequence recognized by AGL TFs, the so-called CArG-box, in their promoter region. As a result, we identified a CArG-box in the promoter region of *PvNIN* and *PvNSP2* (Table 1). We then determined the expression level of these two genes in 2-day-old non-inoculated transgenic roots. Our data indicated that the basal expression level of *PvNSP*2 was similar in the control (EV) and modulated (RNAi_*AGL* or OE_*FUL* roots). However, the gene expression level of *PvNIN* in non-inoculated control EV roots (0.023 ± 0.003) was significantly decreased in RNAi_*AGL* roots (0.001 + 7 – 0.0001) while it significantly increased in OE-*FUL* roots (0.052 ± 0.003). This data supported our hypothesis proposing that PvAGL TF might be implicated in *PvNIN* regulation.

**Table 1:** Identification of CArG-box sequences in *P. vulgaris* early symbiotic genes.

**Gene name**	**Gene ID**	**CArG sequence**	**Position relative to the initiation codon**
*PvNIN*	Phvul.009G115800	CCTTTATAGG	−9166/−9157
*PvNSP2*	Phvul.009G122700	CCATATATGG	−3736/−3727
*PvRIC1*	Phvul.005G096901	CCAAAAAAGG	−16998/−16989
*PvTML*	Phvul.001G094400	CCATTTATGG	−718/−709
		CCAAATTTGG	−7715/−7706
		CCATTAAAGG	−8099/−8090

### Effect of PvAGL TF in Root and Nodule Development of Common Bean in Symbiosis With *R. etli*

We assessed if the affection of rhizobia-infection and early symbiotic gene expression in composite plants with modulation in *PvAGL*-gene expression results in altered root and nodulation phenotype.

**Figure 7:**
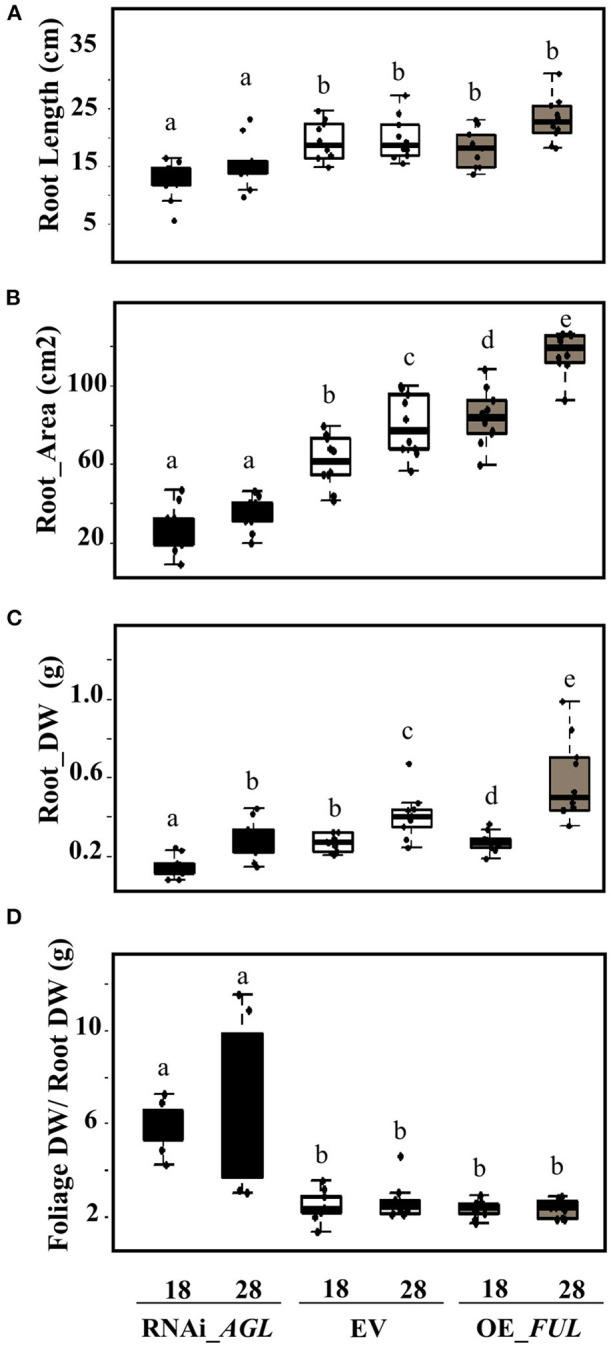
Root phenotype of *R. etli*-inoculated composite plants expressing the RNAi_*AGL*, or OE_*FUL* construct compared with EV. **(A)** Root length, **(B)** area, **(C)** dry weight (DW), and **(D)** foliage DW /root DW ratio were determined at 18 dpi and 28 dpi as indicated. Data shown were obtained from 10 biological replicates per condition. The horizontal box side represents the first and third quartile in box plots while the outside whiskers the minimum and maximum values. Different lower-case letters from each set of values indicate a significant difference according to one-way analysis of variance (ANOVA) (*p*-value < 0.001).

An evident decrease in the transgenic roots formed, and the root growth was observed in *PvAGL*-silenced composite plants (Figures 4B,C). Analysis of root architecture revealed decreased root length, area, and biomass of RNAi_*AGL* roots compared with control (EV) roots, both at 18 and 28 dpi (Figures 7A–C). In agreement, the foliage/root ratio was increased in RNAi_*AGL* composite plants (Figure 7D). Contrastingly, plants transformed with OE_*FUL* showed an adequate formation and root architecture of transgenic roots (Figures 4B,C). In addition, the roots overexpressing *PvFUL*-like showed increased root length, area, and biomass compared with EV roots at 28 dpi (Figures 7B,C).

**Figure 8:**
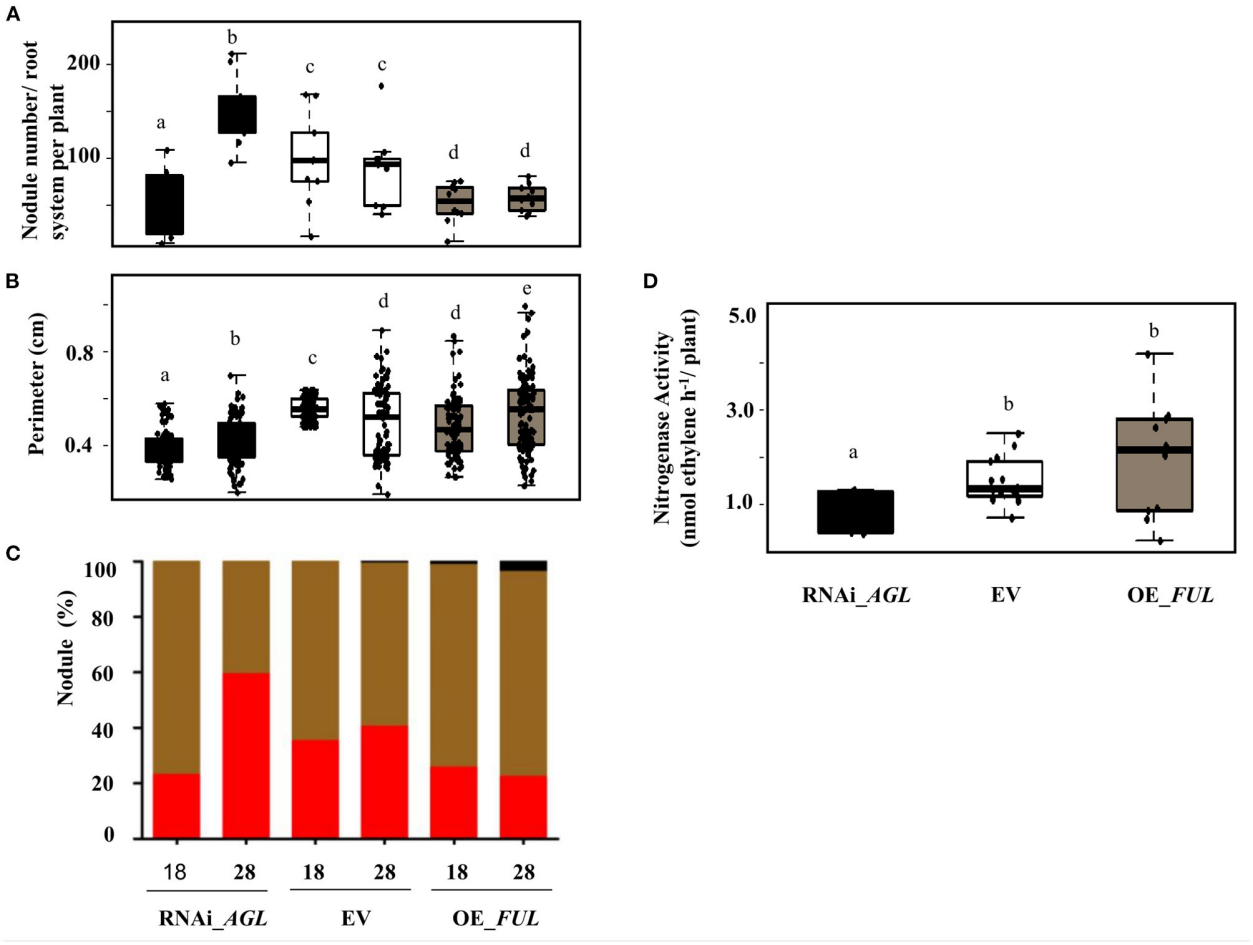
Nodulation phenotype of *R. etli*-inoculated composite plants expressing the RNAi_*AGL*, or OE_*FUL* construct compared with EV. Nodule parameters were determined at the indicated dpi. **(A)** Nodule number from the whole root system per plant was obtained from 10 biological replicates per condition. **(B)** Nodule perimeter was calculated using the ImageJ program from 50 different nodules obtained from three to five plants per condition. **(C)** Based on their perimeter values, nodules were classified as small (0.2–0.5 cm; red bar), medium (0.51–0.75 cm; brown bar), or large (>0.76; black bar), the percentage of nodules from each category is shown for each condition. **(D)** Nitrogenase activity was determined at 28 dpi using the acetylene reduction assay (ARA). Data were obtained from 10 biological replicates per condition. Different lower-case letter columns indicate a significant difference according to one-way analysis of variance (ANOVA) (*p*-value < 0.001).

**Figure 9:**
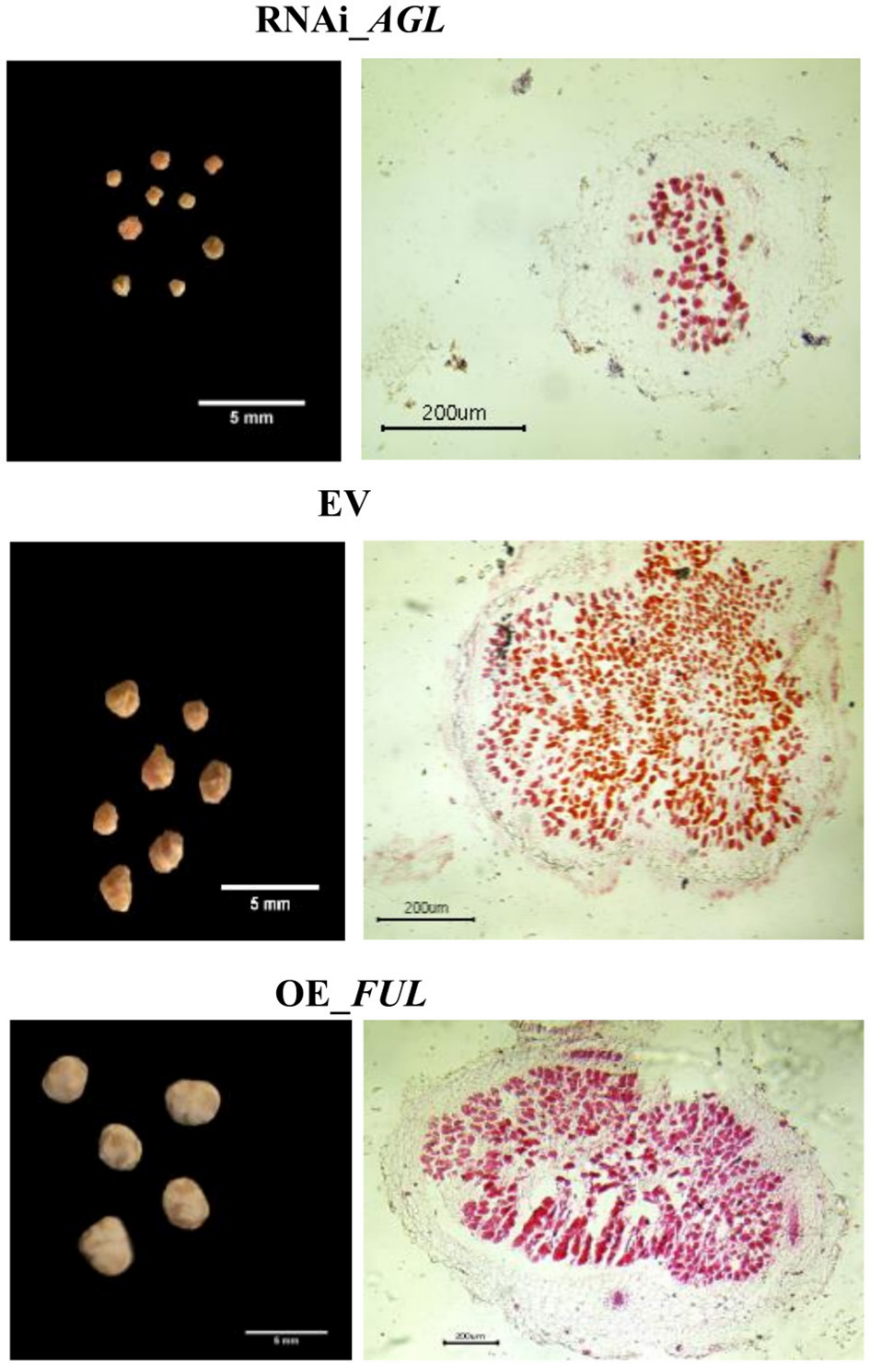
Mature transgenic nodules expressing the *AGL*_RNAi or OE_*FUL* constructs compared with EV. Nodules were excised from transgenic roots at 28 dpi. Left side: representative images of the most abundant type of nodules from each condition. Right side: Transversal sections of mature nodules from each condition stained with safranine and visualized by microscopy. Representative images are shown.

Regarding the nodule phenotype, we observed that compared with EV plants, RNAi_*AGL* composite plants formed fewer nodules at 18 dpi, but at a later stage (28 dpi), their nodule number increased (Figure 8A). A high proportion of small nodules were observed in RNAi_*AGL* transgenic roots at 28 dpi (Figures 8B,C). However, each plant's nodule dry weight per root system was similar among the RNAi_*AGL*, OE_*FUL*, and EV plants. Microscopic image of RNAi_*AGL* nodule sections revealed defective, small nodules with decreased number of infected cells (Figure 9). By contrast, compared with EV plants, OE_*FUL* forms fewer nodules but with increased size (Figures 8A,B). A high proportion of medium and large size nodules was observed in OE_*FUL* plants (Figures 8C, 9). The OE_*FUL* healthy nodules showed a full infected zone (Figure 9).

Nodule function was then evaluated by determining nitrogenase activity through ARA. In agreement with their smaller size and fewer infected cells (Figures 8, 9), RNAi_*AGL* nodules displayed a significantly lower nitrogenase activity compared with that from EV or OE_*FUL* nodules (Figure 8D).

### Effect of PvAGL TF in the AON of the Common Bean – *R. etli* Symbiosis

Knock-out mutants from different legume species, defective in the shoot nodule autoregulation receptor kinase (*NARK*, in common bean and soybean) present a characteristic hypernodulation or supernodulation phenotype; but the nodules formed, in higher number, are ineffective, white and small (Reid et al., 2011). This phenotype resembles the one observed in composite common bean plants silenced in *PvAGL* genes (Figures 8, 9). Thus, we analyzed if RNAi_*AGL* composite plants are altered in the root components of the AON. As shown in Figure 6G, the expression level of the *PvRIC1* gene was very low in *R. etli*-inoculated roots expressing the RNAi_*AGL* construct. This correlates with the low expression of *PvNIN* (Figure 6C), known to be the transcriptional regulator of *RIC* genes (Soyano et al., 2019; Roy et al., 2020). In addition, the *TML* gene, encoding an AON negative regulator of further nodule formation, also shows diminished expression in RNAi_*AGL* transgenic roots (Figure 6H). By contrast, the roots overexpressing *PvFUL-*like formed a lower number of medium or large nodules compared with EV (control) plants (Figures 8, 9) and showed higher expression of *PvNIN* and *PvTML* (Figures 6C,H). Furthermore, we identified CArG-box sequences repeated three times in the *PvTML* promoter region (Table 1); however, the expression level of *PvTML* in non-inoculated transgenic roots was similar to EV silenced and overexpressing roots. Taken together, these results indicate a positive effect of *PvAGL* genes in the AON process of the rhizobia N-fixing symbiosis, possibly exerted through the interaction with *PvNIN*, relevant for the regulation of this process.

## Discussion

In plants, the *MADS*-box/*AGL* genes constitute a large TF family with diverse and essential biological functions to regulate the development of almost every plant organ. Phylogenies of plant *MADS*-box genes have provided information for studies of evolution and developmental genetic pathways (Alvarez-Buylla et al., 2000a; Liljegren et al., 2000). In this work, we present a phylogeny of *MADS*-box genes from Arabidopsis and the legumes: common bean, soybean, Medicago, and Lotus. Initial research of Arabidopsis *MADS*-box genes focused on floral development, and it gave rise to the so-called ABCDE model of floral organ specification (Smaczniak et al., 2012). The phylogenetic tree (Figure 1) indicates the clades of Arabidopsis homeotic genes from A, B, C, D, or E classes that group *MADS*-box genes from the four legumes analyzed. Data from legume GEAs generally indicated a high expression of such legume genes in flowers. For example, the clade, including the class A *AP1* gene from Arabidopsis, involved in specifying sepals and petals (Mandel et al., 1992), also includes genes from the four legume species highly expressed in flowers. However, its expression in specific flower tissues, i.e., sepals and petals, has not been documented. This agrees with previous knowledge indicating that Arabidopsis *MADS*-box genes that form a monophyletic clade share similar tissue expression and likely share regulatory function (Alvarez-Buylla et al., 2000a).

On the other hand, the phylogenetic analysis suggested that the evolution of the MADS-domain family has involved a simultaneous functional diversification in vegetative and reproductive structures (Alvarez-Buylla et al., 2000a; Liljegren et al., 2000). Examples of such diversification were found in the class B genes represented by the *AP3* and *PISTILLATA* (*PI*) gene clades (Jack et al., 1992; Goto and Meyerowitz, 1994). The legume genes grouped in the *AP3* clade include the soybean genes Glyma04G245500 that, according to GEA data, is expressed in flowers and root; and Glyma.06G027200 (*NMH7*) gene that is highly expressed in roots and nodules (Ma et al., 2019). According to the GEA data, the PI clade includes the Medicago Medtr1g029670 gene that, in addition to flower, is highly expressed in nodules.

Based on data from Arabidopsis and legumes gene expression atlas, we analyzed the expression pattern of seven genes from five clades that include seven Arabidopsis *MADS*-box genes known to be important regulators of root development (Alvarez-Buylla et al., 2019; Figure 1). These genes are: *ANR1* that is a key determinant for developmental root plasticity and has a regulatory role in nutrient response through controlling lateral root elongation in response to nitrate (Gan et al., 2005); *XAL1* that plays a principal role during root development, possibly regulating the expression of genes that are components of the cell cycle (Garcia-Cruz et al., 2016); *XAL2*, another relevant gene for Arabidopsis root development, that controls auxin transport via *PIN* (Garay-Arroyo et al., 2013; Alvarez-Buylla et al., 2019); *AGL19* that is expressed in the columella, lateral root cap and epidermal cells of the meristematic region of the primary and lateral root tips (Alvarez-Buylla et al., 2000a); *AGL17* gene that seems to be a lateral root cap marker in the root tip (Burgeff et al., 2002); *AGL21*, which is highly expressed in lateral root primordia and it has a punctual expression in the primary root meristem (Alvarez-Buylla et al., 2019) and *AGL16* gene that is expressed at relatively high levels in the Arabidopsis root quiescent center (Nawy et al., 2005) and shows an intermediate expression level in phloem, xylem and procambium o the root mature zone (Alvarez-Buylla et al., 2019). Evident variations in the expression pattern from seven plant organs were observed among Arabidopsis and the legume *MADS*-box genes grouped in each clade (Figure 2). Such variations may be related to the different ages/conditions of the different organs from the consulted database from each plant species. For example, data for *AGL* genes expression in leaf were from 21-day-old common bean leaves, Gm, 28 days old for Medicago, 42 days old for Lotus, and young leaves for Arabidopsis consulted gene expression atlas. However, all the legume *MADS*-box genes from analyzed clades showed expression in roots, ranging from 9 to 100% compared with the level of expression in other organs. Notably, all the legume genes, except *P. vulgaris* (Phvul.002G143866, Figure 2D), were also expressed in legume nodules (7 to 74%), developed from roots inoculated with rhizobia.

Recent research in Lotus and Medicago has demonstrated that legumes co-opted a lateral root developmental program, from Arabidopsis, to control nodule organogenesis (Schiessl et al., 2019; Soyano et al., 2019). The case of study in these reports are the legume orthologs of the Arabidopsis *ASYMMETRIC LEAVES 2-LIKE 18/LATERAL ORGAN BOUNDARIES DOMAIN 16a* (*ASL18/LDB16a*) gene. In Arabidopsis, the ASL18/LBD16a TF is involved in establishing the asymmetry required for divisions of the pericycle founder cells that produce lateral root primordia (Goh et al., 2012). In legumes, the nodules are lateral root organs that initiate from the inner root layers in response to rhizobial perception. In contrast, lateral roots emerge from predefined founder cells as an adaptive response to external stimuli. Thus, despite differential induction, lateral roots and nodules share developmental programs through *ASL18/LBD16a* (Schiessl et al., 2019; Soyano et al., 2019; Shahan and Benfey, 2020). Thus, the master regulator for legume symbioses, the NIN TF, is essential for recruiting the lateral root and nodule organogenesis developmental programs. In Medicago, cytokinin-induction of *NIN* allows induction of this program during nodulation through activation of *ASL18/LBD16a* that promotes induction of auxin-responsive gene and auxin biosynthesis (Schiessl et al., 2019). In Lotus, *NIN* regulates the *ASL18/LBD16a* and *NUCLEAR FACTOR-Y* (*NF-Y*) genes that genetically interact during nodule development (Soyano et al., 2019). On this basis, research presented in this work about root/nodules *PvAGL* TF genes (namely, *PvFUL*-like, *PvXAL1*-like, *PvAGL24*-like, *PvSOC1*-like, *PvSVP*-like, and *PvAGL16*-like) led us to propose that Arabidopsis *AGL* TF that regulate root development have been co-opted by legumes to control, both, root and nodule development. However, further experimentation is needed to confirm this hypothesis.

The negative effect of *PvAGL* gene silencing in root development was evident in non-inoculated and *R. etli* inoculated plants. These roots showed delayed transgenic roots after *A. rhizogenes* infection and reduced root growth (Figure 4). Furthermore, the inoculated RNAi_*AGL* plants showed altered root architecture evidenced by diminished root length, area, and biomass (Figures 4, 7). Thus, orthologs of Arabidopsis *AGL* genes that regulate root development have a similar function in common beans.

The gene silencing of root/nodule *PvAGL* genes did not affect the root hair development or density (Supplementary Figure 6). However, it negatively affected rhizobial infection of common bean roots, perhaps because due to ineffective chemical communication between the symbionts, something that was evidenced by a significant reduction in the rhizobia-induced effective deformed root hairs that was concomitant with a significant decrease in the expression level of early symbiotic genes essential for rhizobial infection (Figures 5, 6). The genes analyzed include *PvFNSII* involved in the synthesis of flavones, the chemical signal of the plant sensed by compatible rhizobia, and genes involved in the infection thread initiation and progression (*PvFLOT2, PvENOD40*, and *PvENOD93*) that act downstream of the TF NSP2 and NIN (Roy et al., 2020). Thus, the effect of PvAGL TF in the transcriptional regulation of early symbiotic genes (Figure 6) could be direct or indirect. In addition, several other genes are known to be involved in the rhizobial infection process, and infection thread formation and progression (Roy et al., 2020) could be down-regulated in RNAi_*AGL* roots and would negatively affect initial symbiotic stages. Future experiments based on transcriptomic approaches would provide evidence about this proposition.

Research in different legumes indicates that a delay in the rhizobial infection process results in a defective activation of the nodule organogenesis program (Oldroyd, 2013; Roy et al., 2020). In agreement, a defective nodulation phenotype (Figures 8, 9) was evident in *PvAGL-*silenced plants affected by a rhizobial infection. The nodule-specific NIN TF is an essential symbiosis regulator expressed in the epidermis and controls rhizobial infection (Schauser et al., 1999; Liu and Bisseling, 2020). NIN is also expressed in the pericycle and is essential for regulating nodule primordia formation in Medicago (Liu et al., 2019). The complex pattern of spatiotemporal regulation exerted by NIN in Medicago requires different upstream *cis*-regulatory sequences. These include a remote (-18 kb) *cis*-element with cytokinin-response elements essential for nodule organogenesis (Liu et al., 2019). In addition, recently, it was shown that *NIN* is expressed in the proximal part of the infection zone in nodules and plays an essential role in the transition from infection to fixation zones for establishing a functional symbiosis (Liu et al., 2021). Our *in silico* analysis of the *PvNIN* promoter region revealed a CArG-box *cis*-element at−9166 bp upstream of the initiation codon (Table 1). It is noteworthy that *PvNIN* expression was reduced in *PvAGL* silenced root as opposite to *PvFUL*-like overexpressing roots both from inoculated and non-inoculated plants, something that supports the hypothesis of its possible direct transcriptional regulation by AGL TF acting as an essential regulator of rhizobial infection and nodule organogenesis in common bean. However, further work is required to identify the *P. vulgaris* gene targets of *PvAGL* transcription regulation in common bean roots and nodules. Experimental approaches demonstrate their specific binding to CArG-box present in gene promoter regions.

The observed increased nodule number with altered nodule morphology and function in RNAi_*AGL* plants (Figures 8, 9) led us to propose that the downregulation of *PvAGL* genes results in an alteration of the AON symbiotic process. Recently it was shown that gibberellins signaling is a key regulator of the AON process in Lotus (Akamatsu et al., 2020). The endogenous gibberellins from Lotus nodules induce *NIN* expression via its gibberellin-responsive *cis*-acting region. NIN directly induces *CLE-RS* genes (*RIC* genes in the common bean) to activate the AON process (Soyano et al., 2014; Akamatsu et al., 2020). We showed a significantly low expression of *PvRIC1* and *PvTML* (Figure 6), a negative regulator of AON, in *PvAGL*-silenced roots/nodules. In addition, we showed that the expression level of *PvNIN*, with CArG-box *cis*-elements in its promoter region (Table 1), is up- or down-regulated both in inoculated as well as in non-inoculated roots, with increased or silenced *PvAGL* expression, respectively. Thus, we propose that PvAGL TF positively regulates *PvNIN* that, in turn, positively regulates *PvRIC1* via gibberellins. The AON is a complex process that includes almost 20 different genes (Roy et al., 2020). Therefore, the down-regulation of the expression of other genes relevant to regulating the AON process in RNAi_*AGL* roots/nodules could not be excluded. Taken together, our results indicate that the downregulation of *PvAGL*s in roots/nodules negatively affects the AON resulting in a higher number of small, ineffective nodules formed.

The possible contribution of legume AGL TF as regulators of the rhizobia symbiosis is scarcely documented. In alfalfa, three *MADS*-box genes expressed in roots and nodules have been reported, though their functional analysis is lacking (Heard et al., 1997; Zucchero et al., 2001; Páez-Valencia et al., 2008). In soybean, one report characterizes the *GmNMHC5, MADS-*box gene that acts as a positive regulator of root development and nodulation, while the study of *GmNMH7* revealed its negative regulation of nodulation (Liu et al., 2015; Ma et al., 2019). Future in-depth studies are needed to find the commonalities and differences among MADS/AGL TF roles from different legumes as regulators of the N-fixing symbiosis.

## Conclusions

This is the first report about the participation of PvAGL TF as positive regulators of the common bean – rhizobia symbiosis. The data presented in this study attest to the relevance of PvAGL TF as positive regulators of several processes of the common bean – rhizobia symbioses such as root development, rhizobial infection, nodule organogenesis/function, and the autoregulation of nodulation. Furthermore, we propose that *PvAGL* control be exerted via interplay with *PvNIN*, the master symbiosis regulator. Certainly, the knowledge of AGL TF as regulators of N-fixing symbiosis in different legume species would be relevant for a future improvement of this relevant process for ecological and economic reasons.

## Data Availability Statement

The original contributions presented in the study are included in the article/Supplementary Material, further inquiries can be directed to the corresponding author.

## Author Contributions

GH and LA conceived and designed the study. LA, MI-A, MR-S, MR, AL, and S-IF performed the experiments. LA and LL performed *in silico* analyses. LA, OV-L, LG, and GH adviced experiments and analyzed data. LA and GH wrote the manuscript. All authors contributed to the critical revision of the manuscript, read, and approved the submitted version.

## Conflict of Interest

The authors declare that the research was conducted in the absence of any commercial or financial relationships that could be construed as a potential conflict of interest.
