# Supplementary figures and images for: Control of the Rhizobia Nitrogen-Fixing Symbiosis by Common Bean MADS-Domain/AGL Transcription Factors
Source: Front Plant Sci. 2021 Jun 7;12:679463. doi: 10.3389/fpls.2021.679463 (PMC8216239; doi:10.3389/fpls.2021.679463)

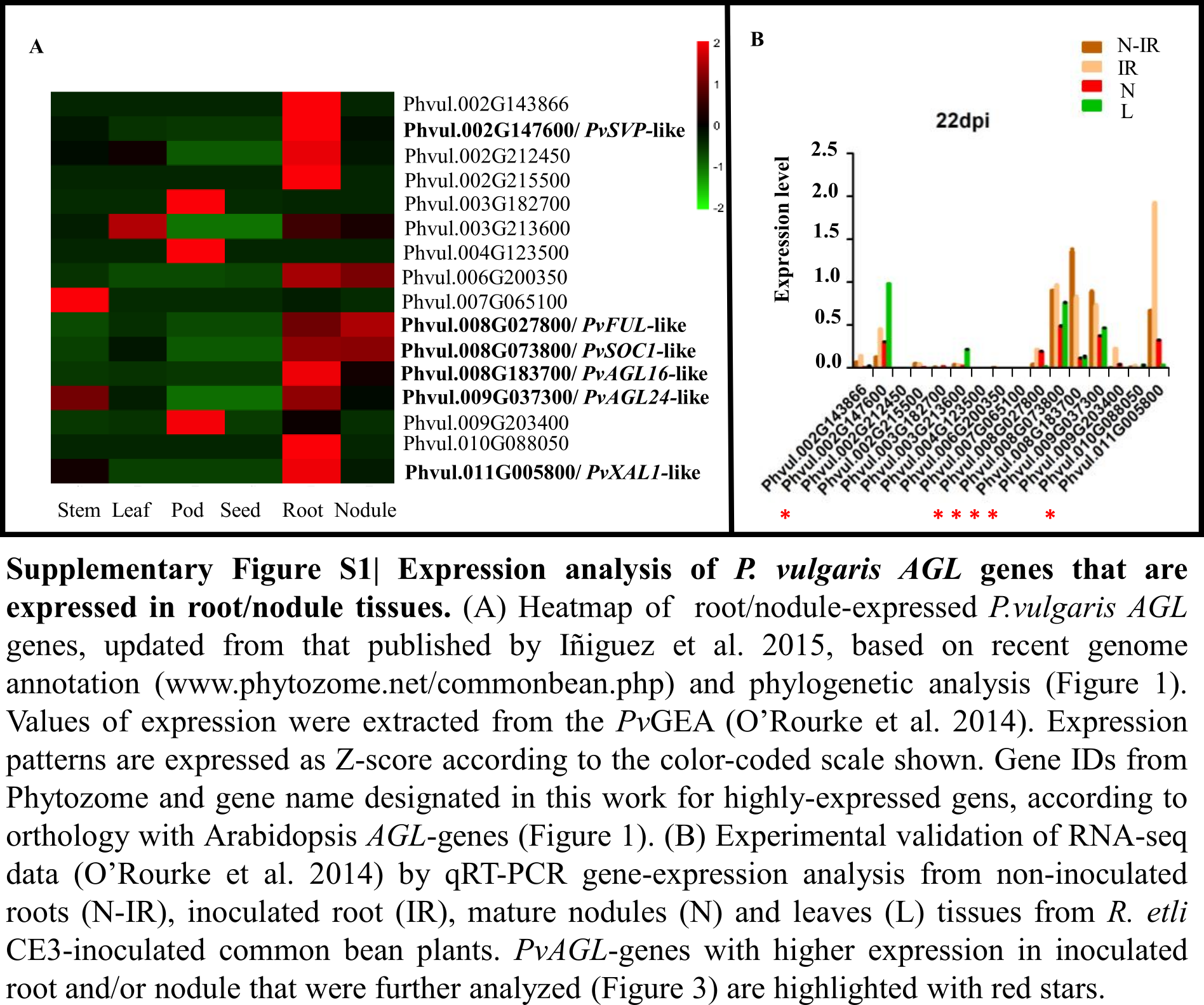

Supplement: Supplementary file 3 [file Image_1.TIF]

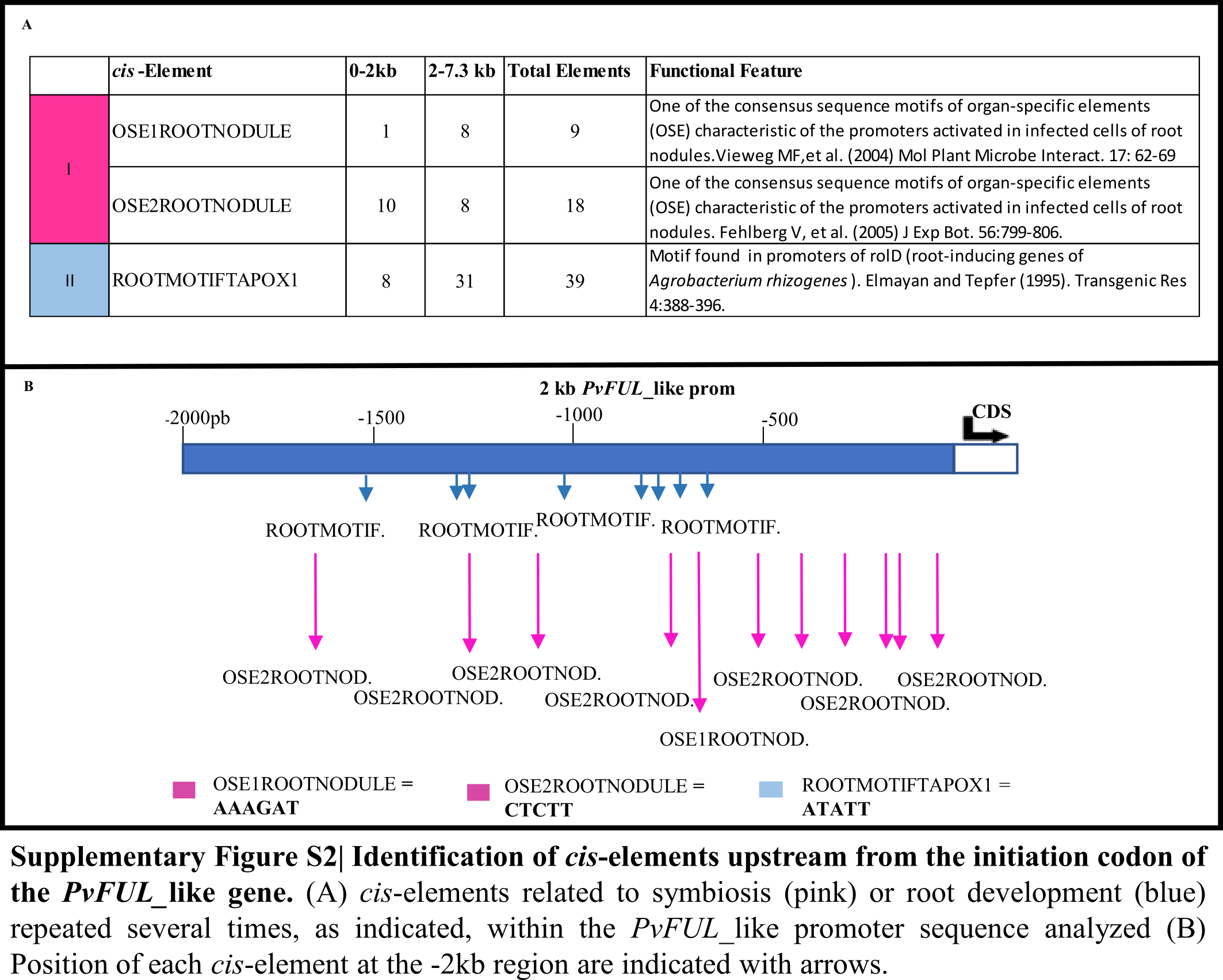

Supplement: Supplementary file 4 [file Image_2.TIF]

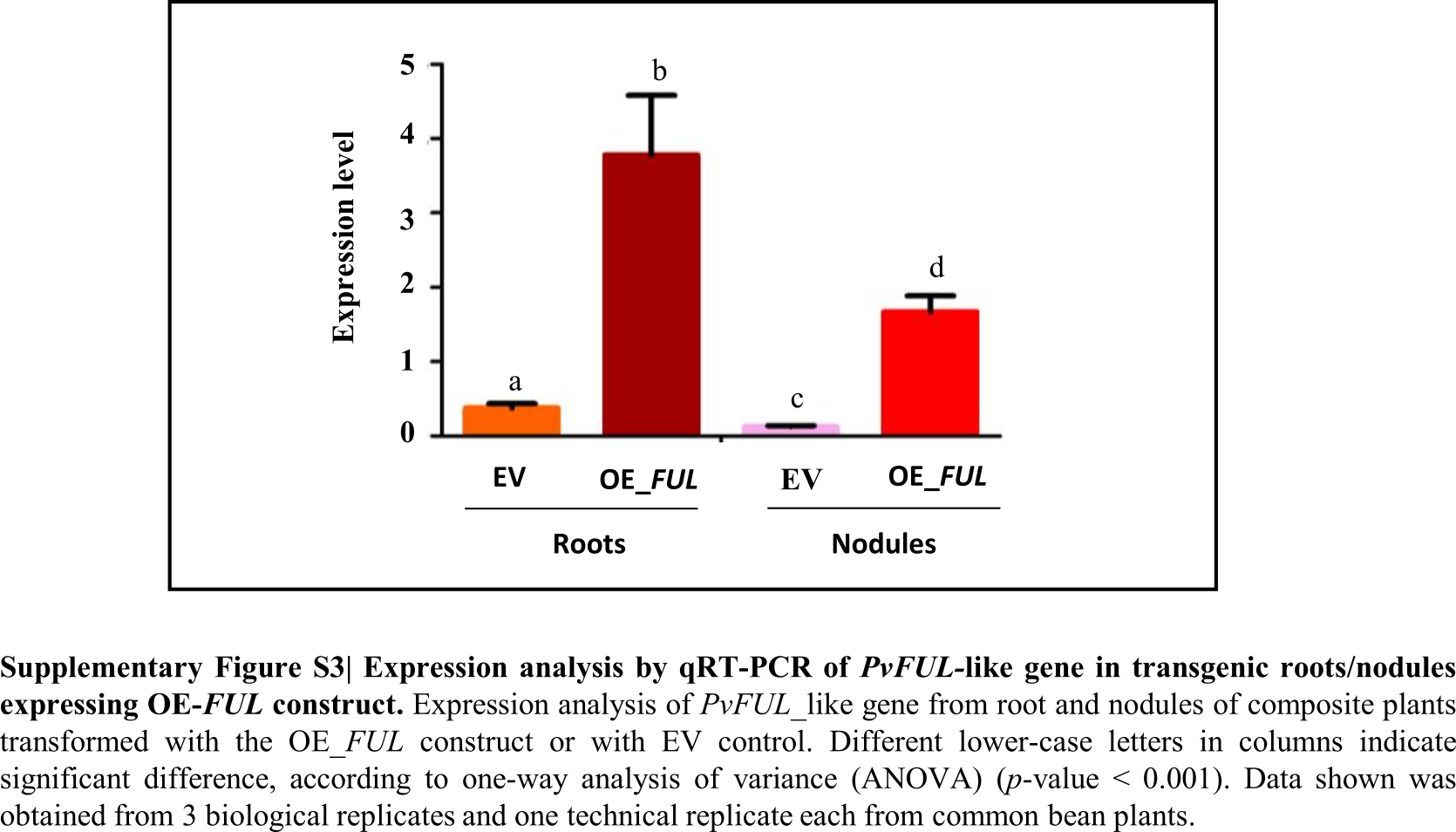

Supplement: Supplementary file 5 [file Image_3.TIF]

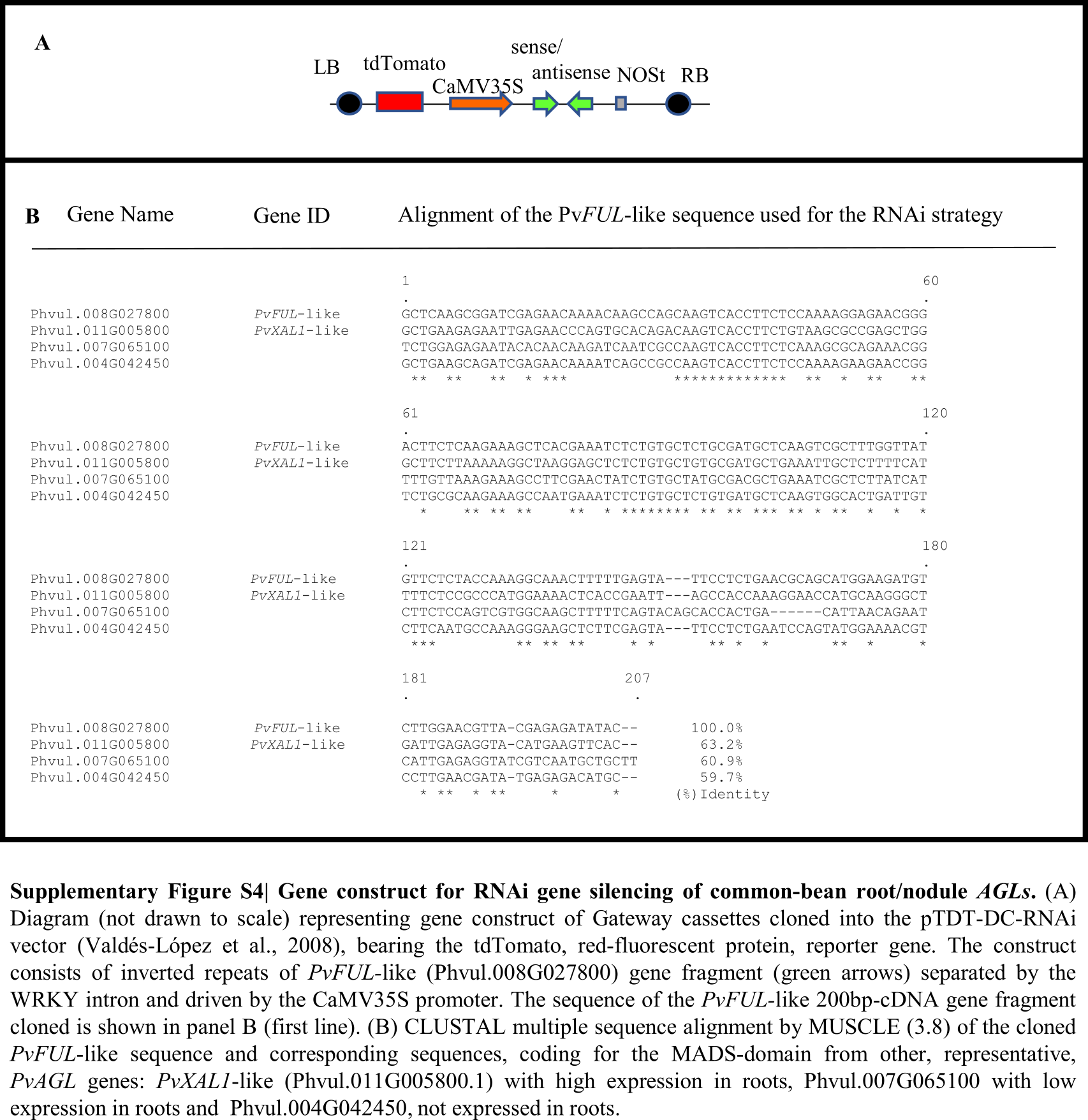

Supplement: Supplementary file 6 [file Image_4.TIF]

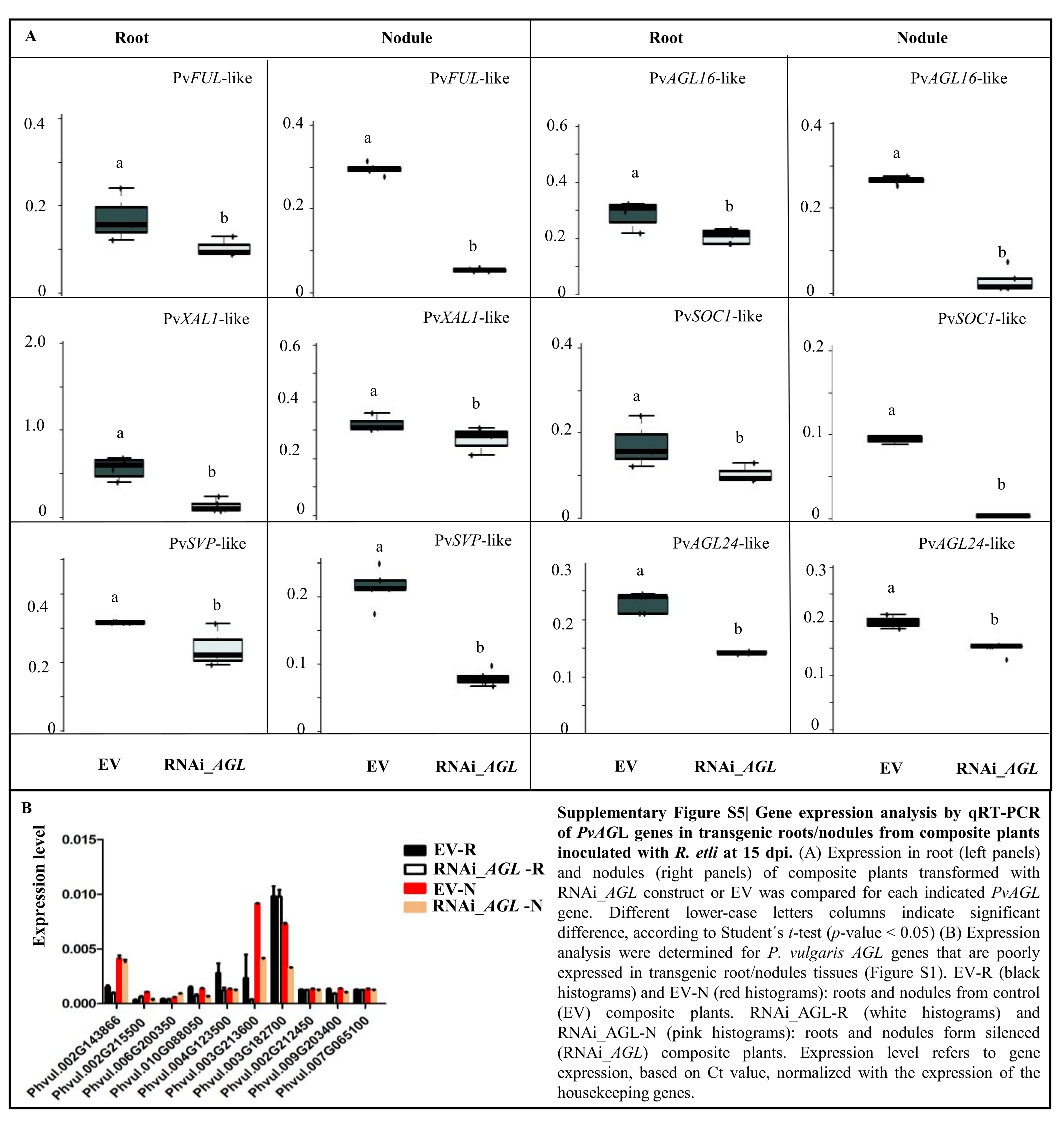

Supplement: Supplementary file 7 [file Image_5.TIF]

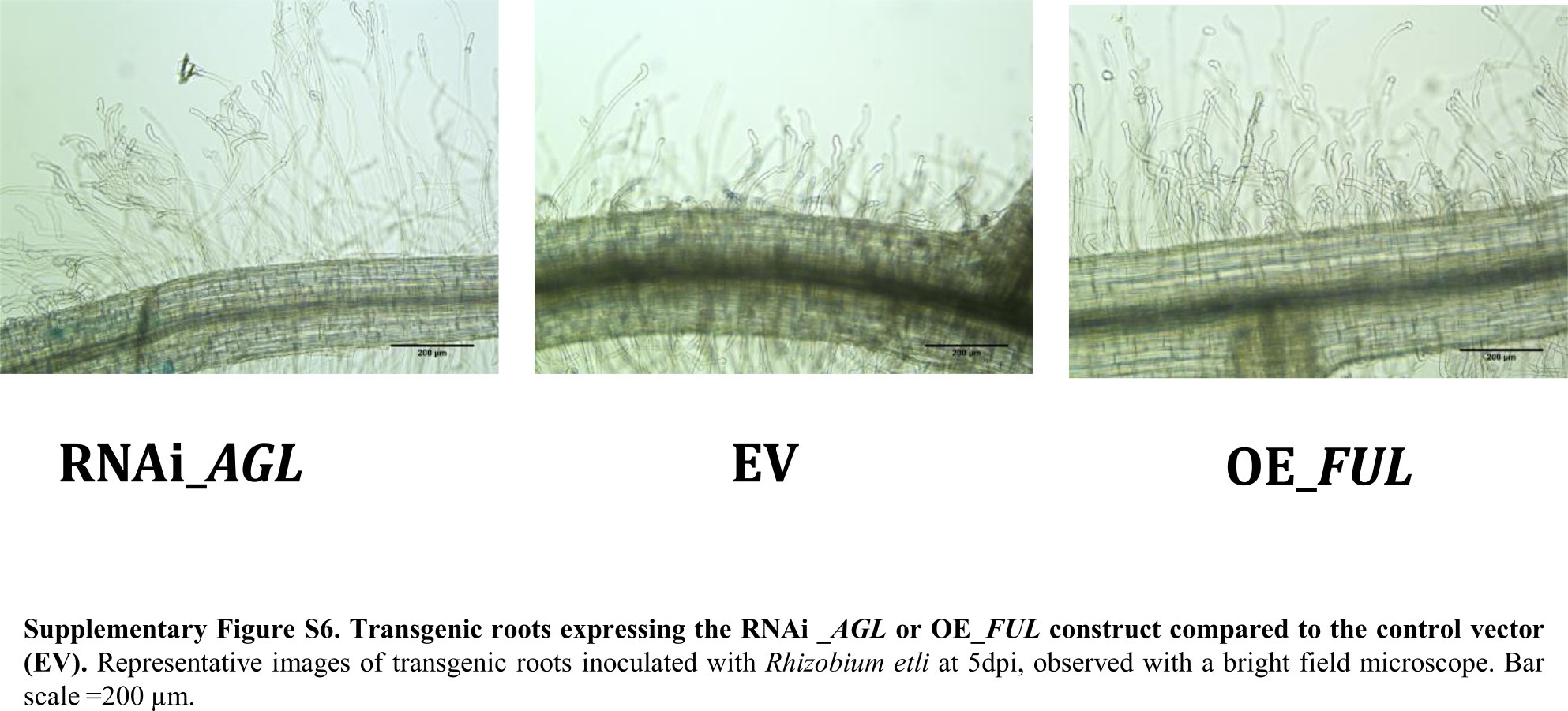

Supplement: Supplementary file 8 [file Image_6.TIF]
